# New tools for high‐throughput expression of fungal secretory proteins in *Saccharomyces cerevisiae* and *Pichia pastoris*

**DOI:** 10.1111/1751-7915.13322

**Published:** 2018-10-05

**Authors:** Mario González, Nélida Brito, Eduardo Hernández‐Bolaños, Celedonio González

**Affiliations:** ^1^ Departamento de Bioquímica Microbiología, Biología Celular y Genética Universidad de La Laguna 38206 La Laguna (Tenerife) Spain

## Abstract

Heterologous protein expression in yeast, mostly in *Saccharomyces cerevisiae* and *Pichia pastoris*, is a well‐established and widely used technique. It typically requires the construction of an expression vector in *Escherichia coli* containing the foreign gene and its subsequent transformation into yeast. Although simple, this procedure has important limitations for the expression of large numbers of proteins, that is, for the generation of protein libraries. We describe here the development of a novel system for the easy and fast expression of heterologous proteins both in *S. cerevisiae* and in *P. pastoris*, under the control of the *GAL1* and *AOX1* promoters respectively. Expression in *S. cerevisiae* requires only the transformation of yeast cells with an unpurified PCR product carrying the gene to be expressed, and the expression of the same gene in *P. pastoris* requires only the isolation of the plasmid generated in *S. cerevisiae* and its transformation into this second yeast, thus making this system suitable for high‐throughput projects. The system has been tested by the extracellular expression of 30 secretory fungal proteins.

## Introduction

The growing number of sequenced genomes among fungi, combined with the use of proteomic and gene expression techniques, has allowed to draw a good picture of the proteinaceous arsenal that these organisms secrete in order to thrive in their environments (McCotter *et al*., 2016). Many of these secretory proteins can be assigned to protein families, providing clues about their roles in fungal biology, but plenty of them have no known function or similarity to well‐characterized proteins. This is the case, as an example, of about one‐third of the 279 proteins experimentally identified in the secretome of the phytopathogenic fungus *Botrytis cinerea* (González *et al*., 2015). The study of a protein's biological function often requires its isolation, and this is frequently achieved by making use of heterologous expression systems. Yeasts, either *Saccharomyces cerevisiae* or, mostly, *Pichia pastoris,* have been proven to be effective expression systems for fungal extracellular proteins, with plenty of successful examples (Ahmad *et al*., 2014; Spohner *et al*., 2015), and various commercial systems are currently available that allow simple and straightforward protein expression and purification. However, all of them require the prior construction in *Escherichia coli* of a plasmid containing the gene of interest (GOI), hampering its implementation for large sets of proteins. High‐throughput strategies have been reported for the expression of foreign membrane proteins in *S. cerevisiae* (Newstead *et al*., 2007; Ito *et al*., 2008; Shiroishi *et al*., 2012), taking advantage of the high homologous recombination rate of this yeast (Joska *et al*., 2014), and a similar system has been devised in *P. pastoris* (Mizutani *et al*., 2011) for plasmid‐borne gene expression, although larger protein quantities are usually obtained in *P. pastoris* when integrative vectors are used (Gellissen *et al*., 2005).

Here, we report novel strategies for the high‐throughput expression of secretory proteins both in *S. cerevisiae* and in *P. pastoris*. Basically, the GOI is amplified by PCR from cDNA and the product is directly transformed into *S. cerevisiae*, resulting in a strain able to secrete the recombinant protein. Tools are provided for expression either by integration of the GOI in the *S. cerevisiae* genome or by placing it in a replicative plasmid. If expression in *P. pastoris* is also desired, then this plasmid can be isolated from the *S. cerevisiae* transformant, digested with a restriction enzyme, and used to transform *P. pastoris*. This new system was developed with the aim of generating yeast collections expressing large numbers of fungal secretory proteins, which could be screened in search of specific protein functions.

## Results

### The *S. cerevisiae* strain YEDIS‐G2: expression of heterologous proteins by integration of the GOI in the genome

**Figure 1:**
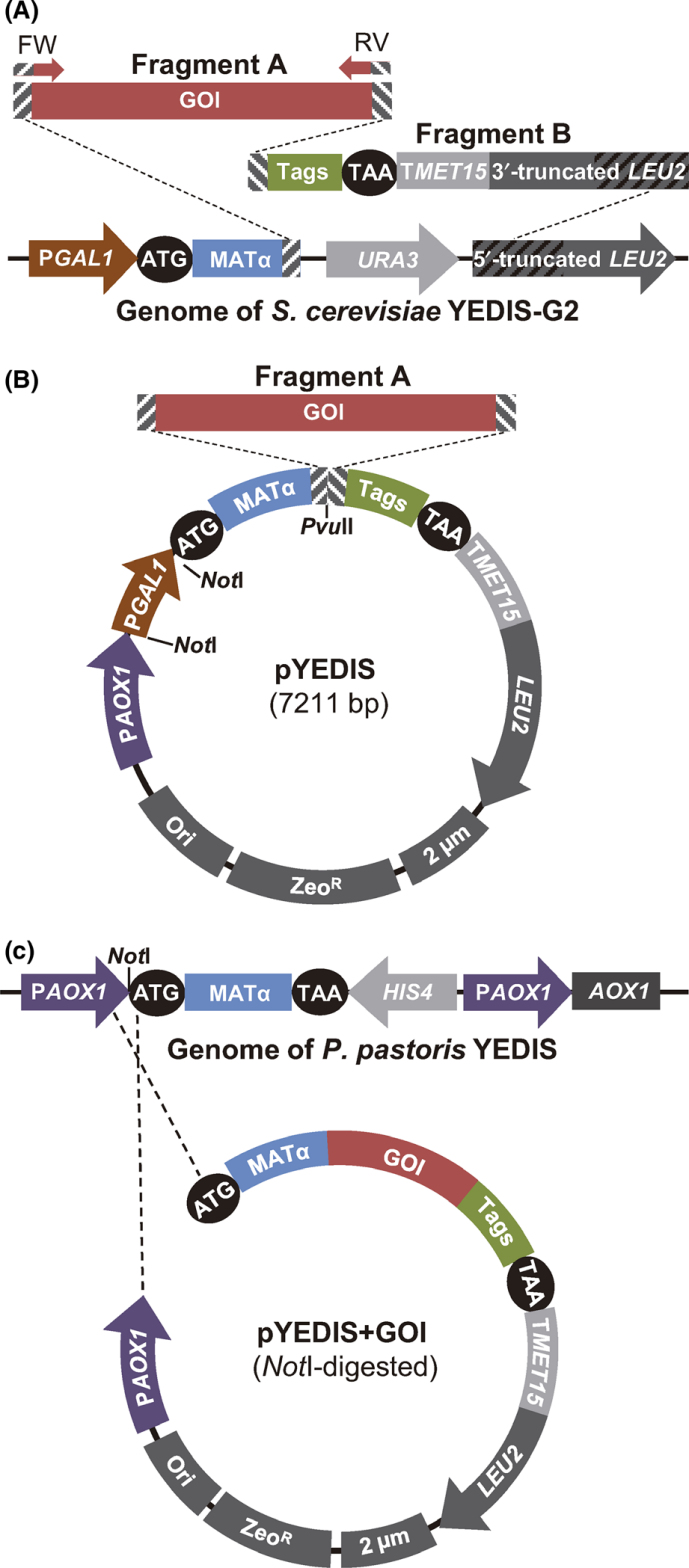
General strategy for the high‐throughput expression of proteins in *Saccharomyces cerevisiae* and *Pichia pastoris*. A. Expression of a gene of interest (GOI) by its integration into the genome of *S. cerevisiae*. A PCR product (Fragment A) carrying the GOI is obtained by PCR from the original organism's cDNA, with primers that introduce tails (dashed rectangles) homologous to the *S. cerevisiae* YEDIS‐G2 genome and to the so‐called Fragment B. When Fragments A and B are co‐transformed into *S. cerevisiae* strain YEDIS‐G2, three homologous recombination events occur that leave the GOI under the control of the *GAL1* promoter (P*GAL* *1*) and fused to sequences coding the α‐factor signal sequence (MATα) and the c‐*myc*, FLAG, and 6xHis tags (Tags). B. Expression of a GOI in *S. cerevisiae* from an autonomously replicating plasmid (pYEDIS). Co‐transformation of Fragment A along with the *Pvu* II‐digested pYEDIS vector results in the generation of the replicative pYEDIS+GOI plasmid. C. Expression of a GOI in *P. pastoris* from a pYEDIS+GOI plasmid rescued from *S. cerevisiae*. Once isolated from *S. cerevisiae* and propagated in *E. coli,* the pYEDIS+GOI plasmid is digested with *Not*I, what results in the release of the *GAL1* promoter. The integration of the digested plasmid into the genome of *P. pastoris* YEDIS results in the expression of the GOI under the control of the *AOX1* promoter. T*MET* *15*:* S. cerevisiae MET15* terminator. Zeo^R^: Zeocin resistance cassette. Ori and 2 μm: *E. coli* and *S. cerevisiae* replication origins respectively. Maps are not in scale.

A new system was developed to allow expression of a heterologous protein in *S. cerevisiae* simply by direct integration of a PCR product carrying the foreign gene into the yeast genome as a single copy (Fig. 1A). The new *S. cerevisiae* strain YEDIS‐G2 was designed for this purpose.

The YEDIS‐G2 strain was generated by genetic modification of the *S. cerevisiae* parental strain BY4741, which displays gene deletions for multiple auxotrophic markers, *URA3* and *LEU2* among them (Brachmann *et al*., 1998). The site for the integration of the GOI was generated at the scar left in strain BY4741 by the deletion of *LEU2*, by transformation with a DNA construct previously obtained by overlap extension PCR (Fig. S1). The transformant DNA contained (i) the *S. cerevisiae GAL1* promoter, a strong promoter often used to direct the expression of heterologous proteins in *S. cerevisiae* (Da Silva and Srikrishnan, 2012), (ii) the *S. cerevisiae* α‐factor signal sequence to deliver foreign proteins to the extracellular medium, (iii) the *URA3* gene for selection of transformants, (iv) a truncated, and therefore non‐functional, *LEU2* region consisting of the last two‐thirds of the CDS plus its own terminator and (v) two border sequences at the ends designed for the integration of the fragment by homologous recombination at the *LEU2* deletion site in strain BY4741 (Fig. S1). The resulting strain was named YEDIS‐G2 (YEDIS = Yeast Expression of the DIssected Secretome), and the correct integration was corroborated by PCR and sequencing of the amplicons (Fig. S1).

In order to express a heterologous gene in *S. cerevisiae* YEDIS‐G2, a PCR product carrying the GOI (Fragment A in Fig. 1A) has to be co‐transformed into the yeast cells along with a DNA construct (named ‘Fragment B’, Fig. 1A) that is always the same regardless of the gene to be expressed.

Primers used for the amplification of the GOI should contain tails (Fig. S2) designed to promote homologous recombination of Fragment A with the end of the α‐factor signal sequence in the YEDIS‐G2 genome, on the one hand, and with the beginning of Fragment B, on the other hand (Fig. 1A). Fragment B is obtained by PCR from plasmid pBSFS+23LEU2 (Fig. S3) and contains in its 5′ end a region that is fused in frame with the 3′ end of Fragment A after recombination occurs, followed by the *c‐myc* and FLAG epitopes, a 6xHis purification tag, a stop codon and the *S. cerevisiae MET15* terminator. Additionally, the 3′ end of Fragment B contains a truncated copy of the *LEU2* gene, consisting of the whole promoter and the first two‐thirds of its CDS (Fig. 1A) and therefore sharing the central one‐third part of the *LEU2* CDS with the truncated *LEU2* copy present in the YEDIS‐G2 genome. Homologous recombination will generate a whole copy of the *LEU2* gene, allowing selection of the transformants (Fig. 1A). Details about Fragment B sequence and the map of pBSFS+23LEU2 are shown in Fig. S3.

Homologous recombination among the two fragments and the genome of *S. cerevisiae* YEDIS‐G2 will leave the foreign gene under the control of *S. cerevisiae GAL1* promoter and fused to the α‐factor signal sequence on its 5′ end, to drive expression and secretion of the recombinant protein, and to sequences adding detection (*c‐myc* and FLAG epitopes) and purification (6xHis) tags (Fig. 1A).

### The pYEDIS vector: an autonomously replicating plasmid for heterologous protein expression in *S. cerevisiae*

It is generally accepted that the increase in the number of copies for a given gene usually results in higher expression. Therefore, a second strategy was developed to increase the copy number of the foreign gene using a 2 μm replicative plasmid for its expression. The novelty of this approach is that it does not require a cloning step in bacteria, since the circular expression vector is generated directly in *S. cerevisiae* by homologous recombination between two fragments that are co‐transformed into the yeast cells (Fig. 1B): a PCR product carrying the GOI, which is identical to the Fragment A discussed in the previous section, and a linearized plasmid (pYEDIS) that carries all the elements contained in the Fragment B described above.

The pYEDIS vector was constructed by fusion of five different fragments (see Experimental procedures) and contains the following elements (Fig. 1B, Fig. S4): (i) the pPICZalpha (Invitrogen, Carlsbad, CA, USA) backbone carrying the *E. coli* origin of replication and the Zeocin resistance marker; (ii) the *S. cerevisiae GAL1* promoter to drive expression of the GOI; (iii) the α‐factor signal sequence, (iv) a sequence identical to the 5′ end of Fragment B, containing the *c*‐*myc* and FLAG epitopes, the 6xHis tail, the stop codon and the *S. cerevisiae MET15* terminator; (v) the *LEU2* marker and the 2 μm origin of replication, for selection and autonomous replication in *S. cerevisiae* respectively; and (vi) the *P. pastoris AOX1* promoter whose necessity will be explained later. pYEDIS also displays a single restriction site for *Pvu*II between the sequences coding for the α‐factor signal sequence and the *c*‐*myc* epitope (Fig. 1B) to allow linearization at a site appropriate for recombination with Fragment A.

After transformation of *S. cerevisiae* BY4741 with the *Pvu*II‐linearized pYEDIS vector along with Fragment A, transformants are selected on SD‐Leu plates. Since Fragment A and the linearized plasmid share homologous sequences at their ends, yeast cells are able to generate a replicative plasmid (pYEDIS+GOI) by homologous recombination after transformation. This new plasmid allows the expression of the GOI under the control of *S. cerevisiae GAL1* promoter and fused with the coding sequences for the α‐factor signal sequence and for the *c‐myc,* FLAG and 6xHis tags (Fig. 1B). Overall, expression of a given GOI by this strategy would only require the amplification of the gene with appropriate primers (Fig. S2) and its transformation into *S. cerevisiae* BY4741 along with the linearized pYEDIS vector.

### *Pichia pastoris* YEDIS: easy transfer of the pYEDIS expression plasmid from *S. cerevisiae* to *P. pastoris*

The use of *S. cerevisiae* in the protocols described above allowed the use of this yeast as an *in vivo* cloning method, thanks to its high homologous recombination rate (Oldenburg *et al*., 1997). However, the yeast *P. pastoris* is well known for its ability to produce higher levels of recombinant proteins than *S. cerevisiae* (Ahmad *et al*., 2014). To take advantage of both systems, a procedure was devised to allow the easy transfer of the pYEDIS+GOI plasmids from *S. cerevisiae* to *P. pastoris*. This transference is possible since the elements necessary for expression in the second yeast were included in the pYEDIS vector (Fig. 1B, Fig. S4): (i) the methanol‐inducible alcohol oxidase 1 (*AOX1*) promoter from *P. pastoris,* to drive the expression of the GOI, and (ii) a Zeocin resistance cassette containing the *Sh ble* gene that serves as a resistance marker in *P. pastoris* and *E. coli*. Besides, the pYEDIS vector has been designed to be able to integrate into the genome of a new *P. pastoris* strain, *P. pastoris* YEDIS, which was generated for this purpose (Fig. 1C).

The *P. pastoris* YEDIS strain was obtained by transformation of *P. pastoris* GS115 (His^*−*^) with a chimeric DNA construct containing the following (Fig. S5): (i) the *AOX1* promoter ending in a newly introduced *Not*I restriction site, (ii) the *S. cerevisiae* α‐factor signal sequence, (iii) a stop codon and (iv) the *P. pastoris HIS4* gene for selection of transformants (see details in the Experimental procedures section). In the resulting strain, there is a copy of the *AOX1* promoter followed by the *Not*I restriction site and the sequence coding for the α‐factor signal peptide (Fig. S5), which is intended to be the site of integration of the *Not*I‐digested pYEDIS plasmids.

To be transferred from *S. cerevisiae* to *P. pastoris*, the pYEDIS plasmids carrying the GOI sequences are simply isolated from *S. cerevisiae*, used to transform *E. coli* for amplification (no screening necessary) and linearized with *Not*I prior to its transformation into *P. pastoris* YEDIS. *Not*I was chosen as its 8‐nucleotide recognition site makes it less probable that the enzyme cuts within the GOI. The enzyme digestion removes the *S. cerevisiae GAL1* promoter (Fig. 1B) and generates a linear DNA fragment with one end homologous to the *AOX1* promoter and the other homologous to the coding sequence for the α‐factor signal peptide (Fig. 1C), therefore favouring the site‐directed integration at the specially designed locus of *P. pastoris* YEDIS. The *Not*I‐*Not*I *GAL1* fragment that also results from the digestion does not need to be removed prior to *P. pastoris* transformation, as this fragment lacks homology with the *P. pastoris* genome. In the rare cases in which *Not*I cuts within the GOI, the whole pYEDIS+GOI plasmid can be amplified with primers facing outwards from the single *Not*I site in pYEDIS (Fig. S4), and the PCR product can be used directly to transform the *P. pastoris* YEDIS strain.

### Expression of secretory *B. cinerea* proteins in *S. cerevisiae* YEDIS‐G2

In order to test the usefulness of the yeast expression systems reported here, several *B. cinerea* secretory proteins previously characterized by our group were selected (Table S1): BcSpl1 (Frías *et al*., 2011, 2014), BcIEB1 (Frías *et al*., 2016; González *et al*., 2017), BcSUN1 (Pérez‐Hernández *et al*., 2017), and the xylanases BcXyn11A (Brito *et al*., 2006; Noda *et al*., 2010), BcXyn11B and BcXyn11C (García *et al*., 2017). These six proteins had also been experimentally identified in various proteomic studies as components of the *B. cinerea* secretome (González *et al*., 2015). The six genes were amplified by PCR from a mix of *B. cinerea* cDNA generated from mycelium grown under different conditions. The primers were designed to amplify the ORF from the first codon after the signal sequence, as predicted by the SignalP 4.1 server (Petersen *et al*., 2011), to the one before the stop codon. The primers also added 5′ and 3′ tails that were required for homologous recombination and integration of the amplicon (Fragment A in Fig. 1A) into the genome of *S. cerevisiae* YEDIS‐G2.

**Figure 2:**
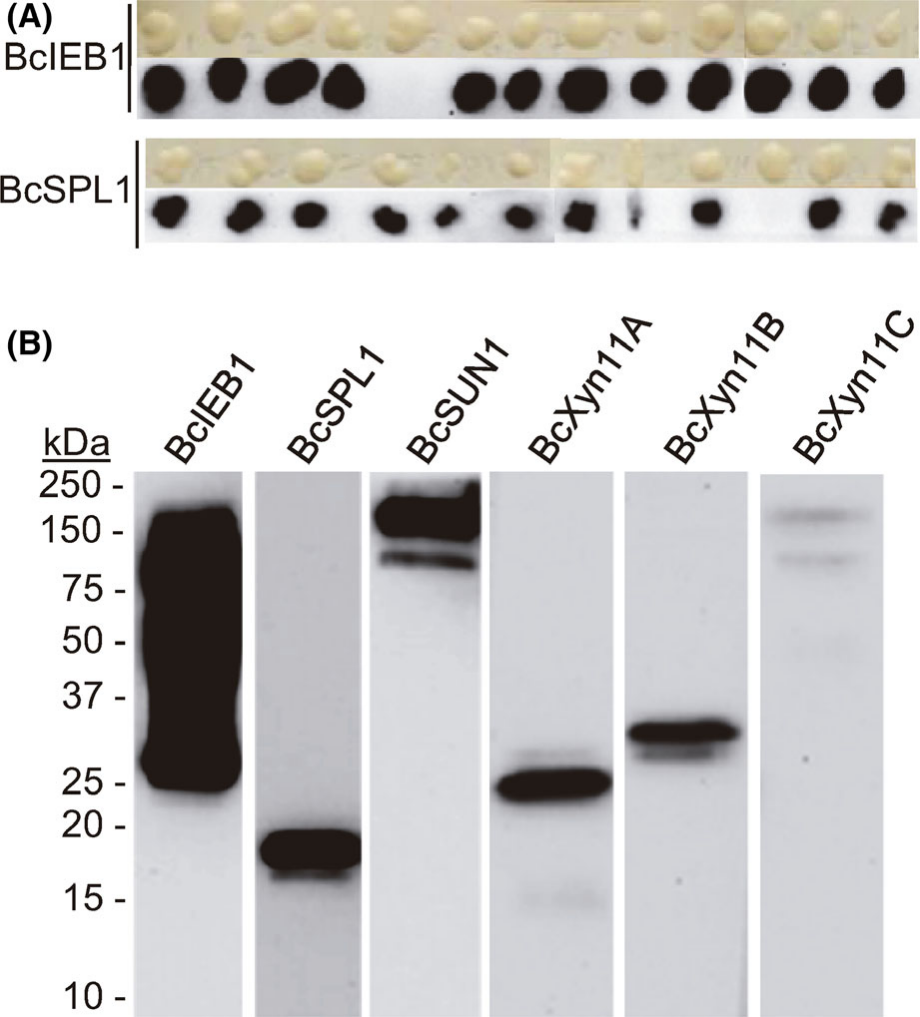
Expression of various *Botrytis cinerea* genes by integration in the genome of *Saccharomyces cerevisiae* YEDIS‐G2. A. Example of colony blot screening for *S. cerevisiae* YEDIS‐G2 transformants expressing the proteins BcIEB1 or BcSPl1. Individual transformants were transferred to nitrocellulose membranes previously placed on YPGal plates, incubated for 2 days at 30°C and then probed with anti‐c‐*myc* antibodies after washing away the cells with water. Upper panels show the colonies on nitrocellulose before washing away the cells, and lower panels show the results of the blot with the antibodies. B. Western blot (anti‐c‐*myc*) of selected transformants expressing the indicated *B. cinerea* proteins. Yeast transformants were grown in YPD and transferred to YPGal medium at an initial OD_600_ = 0.4. Proteins loaded to the blot were precipitated from 1.5 ml of culture supernatant after 24 h of induction with galactose.

Each PCR product was mixed with Fragment B and transformed into *S. cerevisiae* YEDIS‐G2. To screen for transformants expressing the recombinant proteins, a colony blot was carried out, in which individual colonies were grown on a nitrocellulose membranes placed on YPGal plates for 2 days, and the membranes were then probed with anti‐*c‐myc* antibodies (Fig. 2A). The results showed that more than 90% of BcSPL1 and BcIEB1, 50% of BcXy11A and BcXyn11C, and 26% of BcXyn11C colonies were able to express the corresponding recombinant proteins (Table 1). The exception was BcSUN1, since only one colony was detected as positive (Table 1). To corroborate the secretion of the recombinant proteins, selected transformants were induced with galactose in liquid culture and the presence of the recombinant protein in the culture medium was tested by Western blot with anti‐*c‐myc* antibodies (Fig. 2B). The recombinant proteins were always detected, although the molecular masses observed in the Western blot did not always match those calculated from the amino acid sequences (Table S1).

**Table 1:** Expression of six *Botrytis cinerea* secretory proteins in *Saccharomyces cerevisiae* and *Pichia pastoris*

*B. cinerea* Gene	Expression in *S. cerevisiae* YEDIS‐G2^a^	Expression in *S. cerevisiae* with the pYEDIS plasmid		Expression in *P. pastoris* with the pYEDIS plasmid
	Positive transformants/total analysed	Positive transformants/total analysed	Amount of protein secreted (μg ml^−1^)^b^	Amount of protein secreted (μg ml^−1^)^b^
*Bcieb1*	24/26	10/10	1.06 ± 0.07	4.29 ± 0.51
*Bcsun1*	1/26	4/110	0.08 ± 0.02	4.65 ± 0.97
*Bcspl1*	24/26	9/10	0.54 ± 0.04	1.73 ± 0.63
*BcXyn11A*	14/25	9/110	N/A^c^	N/A^c^
*BcXyn11B*	11/42	8/10	0.43 ± 0.03	0.47 ± 0.04
*BcXyn11C*	4/7	60/110	0.1 ± 0.02	0.73 ± 0.23

a Transformation of strain YEDIS‐G2 occurs by integration of a single copy of the foreign gene in the *S. cerevisiae* genome.

b Estimation by Western blot (anti‐*c‐myc*) for 3 (*S. cerevisiae*) or 2 (*P. pastoris*) independent transformants. Western blot signal obtained for 1–2 ml of culture media (after induction of the genes) was compared to that of a known amount of pure BcSpl1 loaded in the same gel.

c Not analysed. Sequence analyses of the plasmids in two chosen clones, from the nine obtained, revealed a sequence not corresponding to the *Bcxyn11A* gene, so expression was not analysed further.

An additional issue to consider in order to use this method in high‐throughput projects is to reduce the cost of primers as much as possible. Therefore, the minimal length of the recombination tails that allows a successful site‐directed integration was studied with the *Bcieb1* gene, by amplifying it from cDNA using primers with recombination tails of 40, 30 or 20 nucleotides (Table S2). As expected, shortening the homologous tails greatly diminished the number of transformants obtained (Fig. S6). However, shorter recombination ends did not affect the frequency of strains expressing BcIEB1 as much. When the primers included 30 or 40 nt recombination tails, more than 90% of the transformants obtained did express the protein, while about 50% of positive colonies were obtained when tails of 20 nucleotides were used (Fig. S6). Taking into account these results, we are now routinely using primers with 30 nt tails for this system.

### Expression of *B. cinerea* proteins in *S. cerevisiae* using the pYEDIS plasmid

**Figure 3:**
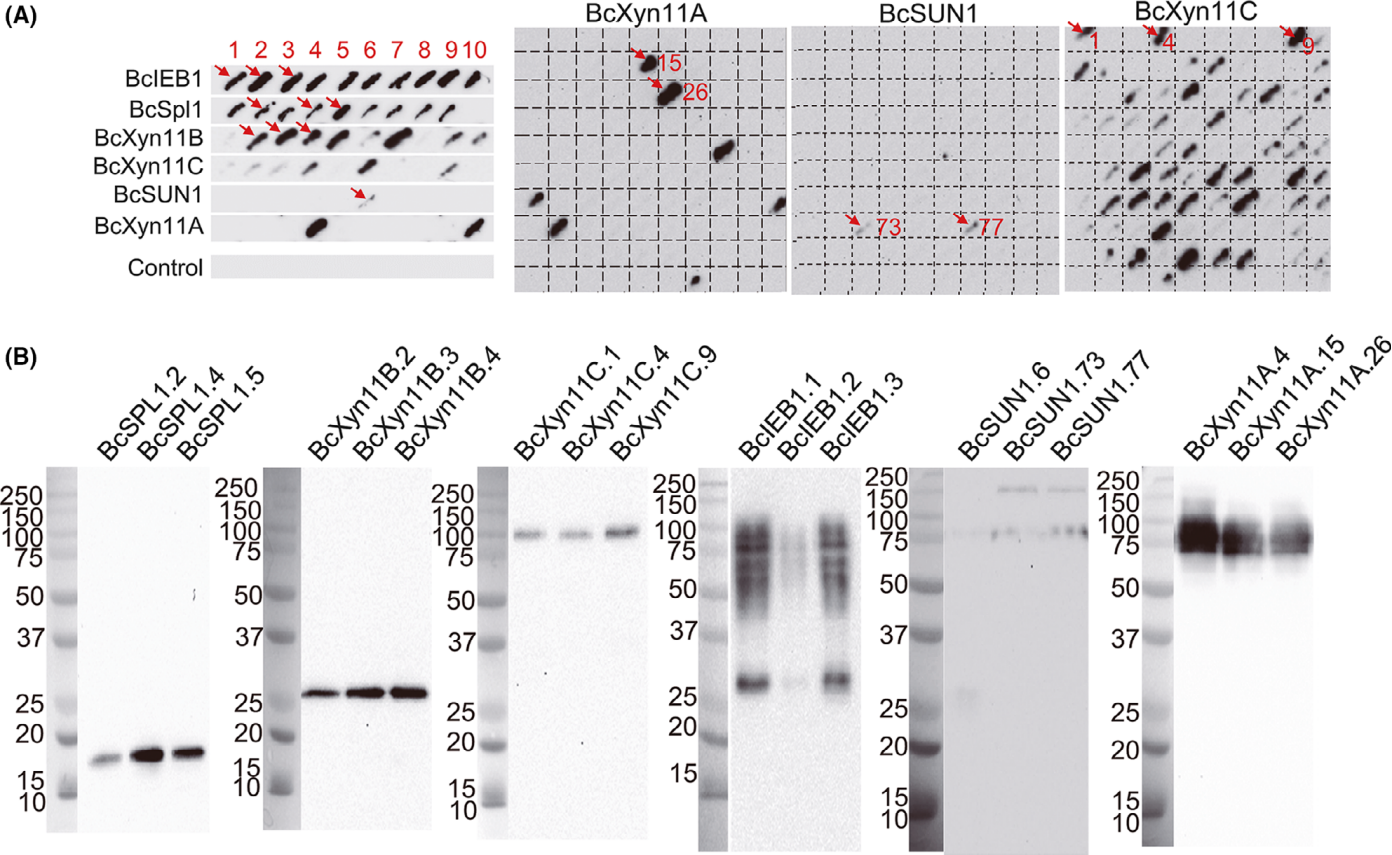
Expression of *Botrytis cinerea* secretory proteins in *S. cerevisiae* using the pYEDIS vector. A. Colony blots (anti‐c‐*myc*) of randomly chosen *S. cerevisiae* transformants screened for the expression of the indicated proteins. Red arrows and numbers serve to identify the clones selected to be further analysed. B. Western blot (anti‐c‐*myc*) analysis for the indicated proteins precipitated from 2 ml of the supernatant from cultures induced with galactose for 24 h. Lanes are labelled with protein names followed by transformant number. Molecular weight markers are shown to the left of each picture along with their sizes in kDa.

The same six *B. cinerea* proteins described above were also selected to check the efficiency of the pYEDIS autonomous replicating plasmid (Fig. 1B) for protein expression in *S. cerevisiae*. In this case, each Fragment A was mixed with the *Pvu*II‐digested pYEDIS vector, and each mix was used to transform *S. cerevisiae* BY4741. The number of transformants that were able to grow without leucine ranged from 1000 to 1200 when Fragment A was present in the transformation mix, while approximately 1500 colonies were obtained when the undigested vector alone was used as positive control. Only 60–70 colonies grew when the transformation was done with the *Pvu*II‐digested pYEDIS alone, therefore suggesting that the vast majority of transformants resulted from the fusion, by recombination, of each Fragment A and the *Pvu*II‐digested pYEDIS vector. To screen for the expression of each recombinant protein, at least 10 colonies were chosen at random from each transformation and analysed by colony blot with anti‐*c‐myc* antibodies (Fig. 3A). More than 80% of the colonies analysed were shown to be positives for the expression of BcIEB1, BcSpl1 or BcXyn11B proteins, 54% for BcXyn11C and less than 10% for BcSUN1 and BcXyn11A (Table 1). As expected, colonies obtained by transformation with the circular pYEDIS vector showed no signal (Fig. 3A).

Three positive colonies from each transformation were further characterized by Western blot analysis with anti‐*c‐myc* antibodies (Fig. 3B), and the results were almost always identical for the three colonies, although the apparent molecular weights observed for the recombinant proteins did not match those expected (Table S1) in every case. In the case of BcIEB1, for example, several bands were obtained, all of them with higher‐than‐expected molecular weight, a phenomenon that has been observed previously for the expression of this same protein in *P. pastoris* and that was attributed to extensive glycosylation (González *et al*., 2017). Size discrepancies were most obvious for BcXyn11A and BcXyn11C, with a difference of about 70 kDa between the observed and expected weights, and in the case of BcSUN1, for which various sizes were observed for the recombinant proteins, ~30 kDa for BcSUN1.6 and ~75 kDa for BcSUN1.73 and BcSUN1.77, while 49 kDa was expected. To find out a possible explanation for these discrepancies, the corresponding plasmids were isolated from the *S. cerevisiae* transformants and sequenced. The sequences of plasmids containing the recombinant *Bcspl1, Bcxyn11B, Bcieb1* and *Bcxyn11C* genes matched exactly the expected ones (data not shown), therefore discarding a modification of the plasmid sequences as the cause of the increase in the apparent size of BcIEB1 and BcXyn11C. In the case of strains BcSUN1.73 and BcSUN1.77, the gene sequences were also as expected, while a deletion of 1074 bp within the *Bcsun1* CDS was observed for the recombinant gene contained in strain BcSUN1.6, which surprisingly conserved the reading frame and thus resulted in a shorter protein still displaying the *c‐myc* epitope (with a theoretical mass of 22.6 kDa). When the plasmid from the *S. cerevisiae* transformant BcXyn11A.4 was sequenced, the insert was completely different from the *Bcxyn11A* gene and coded for an unknown protein with no similarity with any *B. cinerea* protein or any protein in the NCBI protein databases, probably due to a contaminant PCR product. Surprisingly, when the plasmids from two of the transformants with the *Bcxyn11A* gene which were negative in the colony blot assay were sequenced, the two showed the correct sequence, indicating that in the case of this xylanase, the correct plasmid did not result in the production of the recombinant protein. Whatever the reasons are for the lack of expression from this particular plasmid, they seem to be related to the fact that the gene is in a replicative plasmid, as this same protein (BcXyn11A) was successfully expressed using the *S. cerevisiae* YEDIS‐G2 strain (Fig. 2) in which the foreign gene integrates into the genome.

Finally, an estimation of the expression levels for the five recombinant proteins in *S. cerevisiae* is shown in Table 1, which was calculated from three independent Western blots with the assumption that the *c*‐*myc* antibodies bind equally efficiently to all the recombinant proteins. Values ranged from 0.1 to 1.0 μg of recombinant protein per ml of culture supernatant, depending on the protein expressed.

### Expression of *B. cinerea* proteins in the *P. pastoris* YEDIS strain

**Figure 4:**
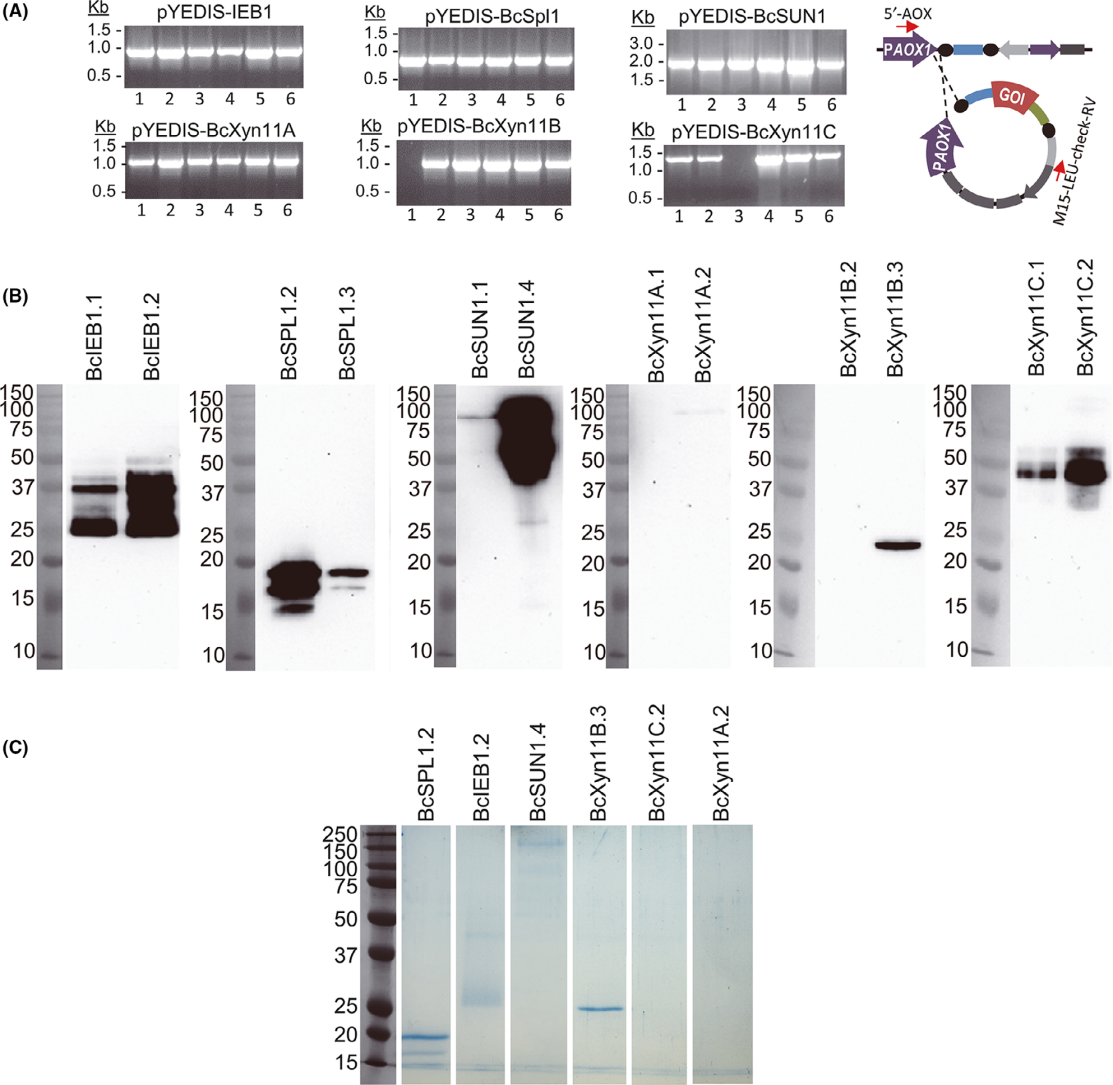
Expression of *Botrytis cinerea* proteins in the *P. pastoris* YEDIS strain. A. Colony PCRs with primers 5′‐AOX1 and M15‐LEU‐check‐RV for six *Pichia pastoris* colonies obtained in the transformation of the indicated plasmids into *P. pastoris* YEDIS. Primer annealing sites are shown in the picture on the right (see details in Fig. 1C). B. Western blot (anti‐c‐*myc*) of the proteins precipitated from 1 ml of the culture supernatant obtained after induction of the indicated strain with methanol for 48 h. C. Trial purification of the recombinant proteins from culture supernatants. Three millilitres of culture supernatant, coming from cultures induced with methanol for 48 h, was subjected to protein purification with nickel magnetic beads. Purified proteins were eluted from the beads and fractionated in SDS‐PAGE, and the gels were stained with Coomassie brilliant blue.

In order to test the easy transfer of the pYEDIS expression plasmids from *S. cerevisiae* to *P. pastoris*, the pYEDIS+GOI plasmids isolated from *S. cerevisiae* described in the previous section were linearized with *Not*I and transformed into *P. pastoris* YEDIS by electroporation. More than 300 Zeocin‐resistant transformants were obtained in every case, and the analysis of six of them by colony PCR (Fig. 4A) showed that most (93.7%) displayed the integration of the foreign DNA at the expected place (Fig. 1C). Two PCR‐positive colonies from each transformation were selected to analyse the production of the recombinant proteins by Western blot, after induction with methanol (Fig. 4B), and the six recombinant proteins were detected in the blots although differences in protein production for clones expressing the same protein were observed. Notably, the same protein that was not expressed by *S. cerevisiae* using the pYEDIS plasmid with the *Bcxyn11A* gene (the one with the correct sequence) was also not detected in the *P. pastoris* YEDIS cultures. Western blots were used to estimate the amount of secreted protein (Table 1), and the results showed that, as expected, the *P. pastoris* transformants usually secrete higher protein quantities than the corresponding *S. cerevisiae* strains.

Since the BcXyn11A recombinant protein was not detected in the supernatants for the *P. pastoris* transformants analysed, whole‐cell protein extracts were obtained and analysed by Western blot to test whether this protein was not produced at all or was produced but not secreted. No signal was observed in any case (not shown), suggesting that the lack of production is due to intrinsic features of the corresponding gene that interfere with gene expression in *P. pastoris* by unknown means, such as premature ending of transcription or translation, or codon bias.

Finally, trial purifications with nickel‐charged magnetic beads were carried out for the six recombinant proteins from 3 ml of culture supernatant, including one of the *P. pastoris* transformants expressing the *Bcxyn11A* gene in spite of the negative Western blot result. As expected, no protein was obtained in the purification of BcXyn11A, but recombinant BcIeb1, BcSpl1, BcSun1 and BcXyn11B proteins could be purified in sufficient amount to be detected on SDS‐PAGE gels (Fig. 4C). However, BcXyn11C was either not purified at all or the quantity recovered was not enough to be detected by staining with Coomassie brilliant blue.

### High‐throughput expression of *B. cinerea* proteins in *S. cerevisiae* using the pYEDIS vector

**Figure 5:**
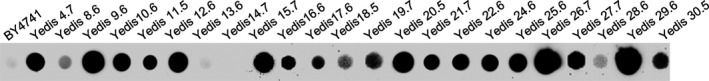
Dot blot analysis of high‐throughput expression of *Botrytis cinerea* proteins in *Saccharomyces cerevisiae* using the pYEDIS vector. Twenty‐three *B. cinerea* genes (see details in Table S3) were expressed by transformation of *S. cerevisiae* BY4741 with the appropriate PCR product (Fig. S7A). One protein‐expressing transformant for each transformation, identified as positive by a combination of colony blot and colony PCR, was used for protein expression trials by induction with galactose in liquid medium for 24 h. Recombinant proteins were detected by dot blot probed with anti‐*c‐myc* antibodies, from 2 ml of culture supernatant. Parental strain (BY4741) was included as negative control.

Once the usefulness of the pYEDIS vector had been tested for the expression of the above six *B. cinerea* proteins in both *S. cerevisiae* and *P. pastoris*, its utility as a high‐throughput system was further explored by attempting the expression of a total of 24 additional *B. cinerea* secretory proteins (Table S3). These proteins were selected because they had been shown to be quite abundant in the secretome in two previous proteomic studies (Espino *et al*., 2010; González *et al*., 2014). All genes were amplified by PCR with primers designed according to Fig. S2 and containing 30 nucleotide tails for recombination. With the exception of two genes, an amplicon with the expected size was obtained at the first attempt (Fig. S7A). Five microlitres of all PCR products, even those for which no product could be detected, was mixed with the *Pvu*II‐digested pYEDIS vector and transformed into *S. cerevisiae* BY4741. All transformations resulted in a number of transformants higher than the control (*Pvu*II‐digested pYEDIS alone), except in the case of Yedis23 in which the number of transformants obtained was similar to the control (not shown). At least 10 different colonies from each transformation were first analysed by colony blot as described before (not shown), and two to four positive transformants were further characterized by colony PCR with primers MATα‐check‐FW y M15‐LEU‐check‐RV to confirm the presence of the GOI (Fig. S7B). With the exception of Yedis23, at least one of the screened colonies was positive in all the transformations. Indeed, in 20 out of 24 transformations, all the colonies analysed were detected as positives by colony PCR. Finally, one positive strain for each transformation was further characterized by inducing the production of the recombinant protein with galactose for 24 h in liquid cultures. The amount of protein produced was then assayed by loading 2 ml of culture supernatant to a dot blot that was probed with anti‐*c‐myc* antibodies (Fig. 5). Although the levels of expression showed a great variation, the results showed that 21 out of 23 could be successfully expressed in *S. cerevisiae*, and remarkably, the whole process took about 2 weeks to be completed.

## Discussion

Phytopathogenic fungi secrete hundreds of proteins during the infection process, many of them with unknown function, and a high‐throughput secretory yeast expression system would be a helpful tool for the characterization of the individual proteins that make up the secretome. In this work, we have designed, constructed and validated a novel system, which allows the expression of a large number of recombinant proteins in two different yeast hosts and in a short time. The main procedures of this system can be summarized as follows: (i) transformation of *S. cerevisiae* with a PCR product (unpurified) carrying the gene of interest, (ii) screening of a few yeast transformants by colony blot and colony PCR to identify clones expressing the heterologous protein, (iii) extraction of plasmid from the selected *S. cerevisiae* strains and amplification in *E. coli* and (iv) transformation of *P. pastoris* with the linearized plasmid and screening of yeast transformants by colony PCR. To achieve the same goal by regular expression systems, it would be necessary to construct two different plasmids in *E. coli*, one for each yeast, prior to the transformation of the two organisms. Even with the use of novel approaches, such as the Gibson assembly method (Gibson *et al*., 2009) used in this work, plasmid construction greatly hampers high‐throughput protein expression in yeast.

Plenty of alternative expression platforms are available for the expression of heterologous proteins in *S. cerevisiae* and *P. pastoris* (Chumnanpuen *et al*., 2016), including various commercial kits and even numerous protein expression custom services. Compared with them, the system we have developed does not add advantages in terms of increased protein production or easier purification of the protein product. Its only and great advantage is a much easier and faster generation of the recombinant strains expressing a given protein in the two yeasts, which allows its application to a large number of genes. Moreover, all the existing protocols for increased protein production in *P. pastoris*, such as the selection of multicopy transformants or improving the expression conditions (Ahmad *et al*., 2014), could be applied to the *P. pastoris* YEDIS strain, since there is no fundamental difference between this strain and other *P. pastoris* expression hosts for the expression under the *AOX1* promoter, other than the way by which the foreign gene integrates into the genome.

During the validation of the expression system reported here, six *B. cinerea* proteins were selected for its expression in both *S. cerevisiae* and *P. pastoris*. All of them were successfully expressed at least in one yeast host. However, the apparent size of the expressed proteins, as observed in SDS‐PAGE, was frequently larger than predicted from the amino acid sequences, and the presence of several protein species of different sizes was not uncommon (see Table S1 for the theoretical expected mass and Figs 2, 3, 4). This is by no means unusual, as *S. cerevisiae* and *P. pastoris* have a tendency to hyperglycosylate secretory proteins (Hamilton and Gerngross, 2007; Cregg *et al*., 2009; Ahmad *et al*., 2014). As an example, the generation of a single new *N*‐glycosylation site in the hen egg white lysozyme, by site‐directed mutagenesis of the corresponding gene, resulted in a 70 kDa increase in the molecular weight of the protein when the mutant gene was expressed in *S. cerevisiae* (Nakamura *et al*., 1993).

As for other expression systems, undesired mutations could potentially be introduced during amplification by PCR of the genes to be expressed, even if high‐fidelity DNA polymerases are used, as is the case of this work. To avoid such problems, it is always possible to sequence the PCR product prior to *S. cerevisiae* transformation, but the best approach would be to sequence the gene in the recombinant plasmid isolated from *S. cerevisiae* and ready to transform *P. pastoris*. By doing so, changes in DNA sequence appearing during transformation and recombination could also be detected.

**Figure 6:**
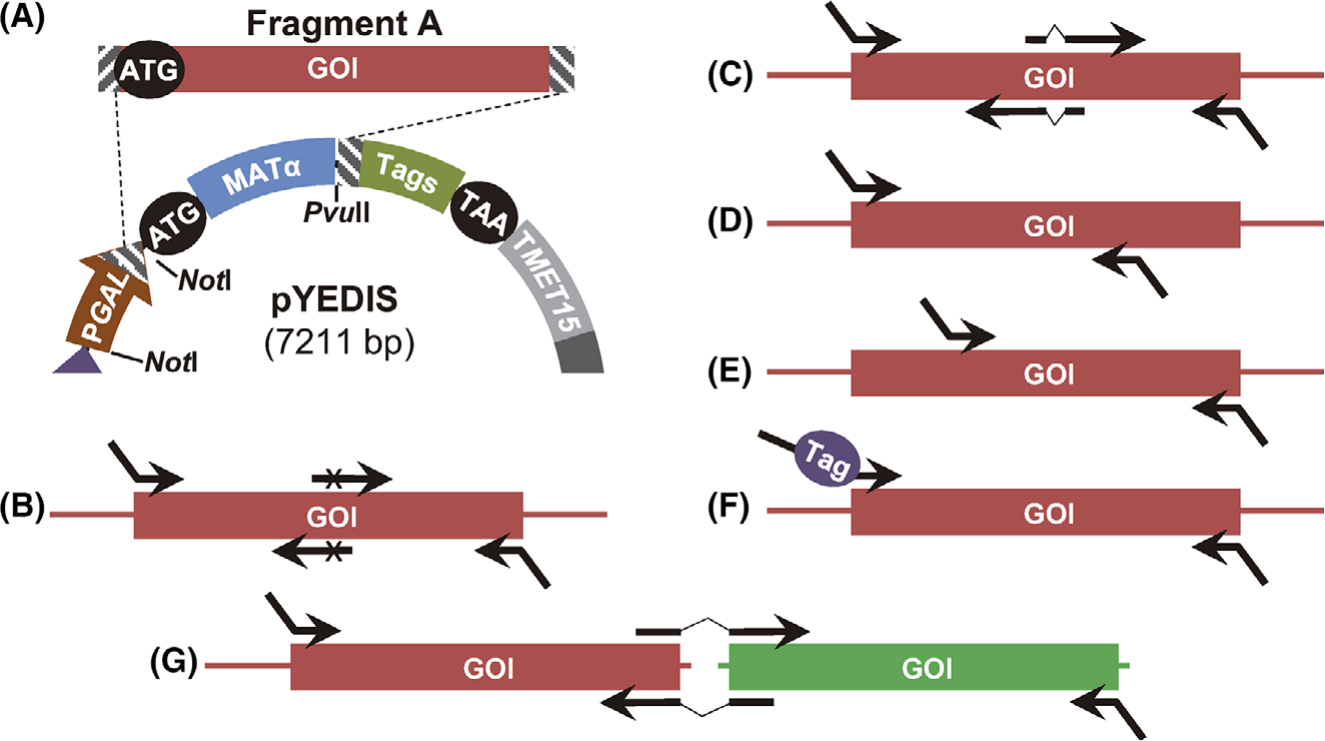
Possible modifications of the YEDIS system to achieve additional goals. A. Modification of the tail of the forward primer allows intracellular expression of the foreign gene. B,C. Amplification of two independent fragments of the GOI with appropriate internal primers allows the introduction of point mutations (x) in the expressed proteins or the generation or internal deletions. D,E. Moving the forward and/or reverse primers would allow the expression of truncated proteins. F. Additional tags can be added to the expressed proteins by adding the coding sequence (Tag) to the desired primer. G. Protein fusions can be expressed by adding a second PCR product to the transformation, as long as it has the appropriate recombination ends.

Although the expression strategy described in this work has been designed for the extracellular expression of proteins, simple modifications would allow its application for other purposes. Intracellular expression in *S. cerevisiae*, for example, could be addressed simply by adding a tail homologous to the end of the *GAL1* promoter, instead of homologous to the end of the α‐factor signal sequence, in the forward primer used to amplify the GOI (Fig. 6A). Inclusion of extra primers complementary to the inner part of the GOI, in order to amplify several independent GOI fragments, may be used for a variety of purposes such as the simultaneous generation and expression of proteins containing site‐directed mutations or proteins with internal deletions (Fig. 6B,C). Truncated versions of proteins could also be produced simply by moving the forward or reverse primers (Fig. 6D,E). Additional small tags could be added to the GOI during expression by simply including the coding sequence in the forward or reverse primers (Fig. 6F), and finally, protein fusions could also be easily generated if an additional fragment with the second protein, displaying the appropriate recombination ends, is added to the transformation (Fig. 6G). All these applications would only require to carry out the PCRs and its direct transformation into *S. cerevisiae*.

## Experimental procedures

### General methods

PCRs were carried out with *Phusion* DNA polymerase (New England Biolabs, Ipswich, MA, USA) if the amplicon was going to be cloned, used for yeast transformation or sequenced, and with *Taq* DNA polymerase in any other case. Primer sequences (Integrated DNA Technologies, Leuven, Belgium) are shown in Table S2, and details about the design of the primers to amplify the genes to be expressed in yeast can be found in Fig. S2. The E.Z.N.A. Yeast Plasmid Kit (Omega Bio‐tek, Norcross, GA, USA) was used for plasmid extraction from *S. cerevisiae*, and plasmid amplification in *E. coli* was done by established methods (Sambrook and Russell, 2001). DNA fragments were sequenced at Secugen S.L. (Spain).

In order to generate the cDNA from *B. cinerea*, equal amounts of mycelium coming from the following four liquid media were mixed: (i) minimal medium with glucose (0.3% Gamborg's B5, 2 mM sucrose, 10 mM K_2_PO_4_, 0.05% Tween‐80, 1% glucose); (ii and iii) the same medium without glucose but supplemented with a dialysis bag containing either tomato or kiwi extract (Espino *et al*., 2010); and (iv) the rich medium YGG (0.5% yeast extract, 0.3% Gamborg's B5, 2% glucose). In all cases, cultures were inoculated with 6 × 10^6^ conidia ml^−1^ and incubated at 20°C for 18 h at 180 rpm. Purification of *B. cinerea* RNA was performed with the RNeasy Plant Mini Kit (Qiagen, Hilden, Germany), and cDNA synthesis was performed using the ProtoScript II First Strand cDNA Synthesis Kit (NEB), as instructed by manufacturers.

Yeast genomic DNA was prepared as Hoffman (1997), and *S. cerevisiae* transformations were carried out as described by Gietz and Woods (2002). For the expression of foreign proteins in *S. cerevisiae,* 5 μl of the PCR mixture was directly used in the yeast transformation (without any DNA purification step), along with 50 ng of the *Pvu*II‐digested pYEDIS plasmid when necessary. Transformants were selected on SD‐LEU or SD‐URA plates (2% glucose, 0.17% YNB without amino acids and ammonium sulfate, 0.16% dropout mix (Sigma‐Aldrich, San Luis, MO, USA, Y1376 or Y1501), 0.5% ammonium sulfate, 1.5% agar). *P. pastoris* transformations were done by electroporation with a Bio‐Rad (Hercules, CA, USA) gene pulser, following manufacturer's recommendations, and transformants were selected on YPDS plates (1% yeast extract, 2% peptone, 2% glucose, 1M sorbitol, 1.5% agar) containing 100 μg ml^−1^ Zeocin.

Induction with galactose of the *S. cerevisiae GAL1* promoter was routinely done by first growing in YPD medium (1% yeast extract, 2% peptone, 2% glucose) until stationary phase and then inducing for 24 h in YPGal (1% yeast extract, 2% peptone, 2% galactose) with an initial OD_600_ of 0.4. Induction of the *P. pastoris AOX1* promoter with methanol was done essentially as instructed by the EasySelect Pichia Expression Kit (Invitrogen) by first growing in BMGH medium and then inducing in BMMH medium for 2 days. Where indicated, proteins in the culture medium were precipitated with methanol–chloroform (Wessel and Flügge, 1984) and resuspended in loading buffer prior to SDS‐PAGE. Intracellular protein fractions from *P. pastoris* were obtained by resuspending the pelleted cells from 10 ml of culture in 200 μl of PBS (137 mM NaCl, 2.7 mM KCl, 10 mM Na_2_HPO_4_, 2 mM KH_2_PO_4_) + 1 mM PMSF and breaking the cells with a FastPrep‐24 homogenizer (MP Biomedicals, Santa Ana, CA, USA) for 30 s at 4 m s^−1^ with glass beads (0.5 mm diameter). Extracts were then mixed with ½ volume of 3× SDS‐PAGE loading buffer, boiled for 10 min, and centrifuged (5 min, 13000 rpm), prior to loading to the electrophoresis gel. SDS‐PAGE (10% acrylamide) was carried out using the Mini‐PROTEAN Electrophoresis System (Bio‐Rad), following manufacturer's instructions, and gels were either stained with colloidal Coomassie brilliant blue G250 (Neuhoff *et al*., 1988) or used for Western blots as previously described (González *et al*., 2014). Dot blots were performed using the manifold SMH48 from SCIE‐PLAS (Cambridge, UK) by loading 2 ml of culture medium directly to a nitrocellulose membrane.

For colony blots, *S. cerevisiae* transformants were transferred to nitrocellulose membranes, previously sterilized by irradiation with UV for 5 min and overlaid on YPGal plates, and grown at 30° until colonies were visible, usually for 24–48 h. The cells were then washed off by rinsing with water, and the recombinant proteins bounded to the membrane were detected with anti‐*c*‐*myc* antibodies following the same protocol as that used in Western blots. DNA templates used in colony PCRs were prepared by transferring the colony to 20 μl of TE buffer, adding glass beads (0.5 mm diameter) to fill about 3/4 of the volume already in the tube, homogenizing in with FastPrep‐24 (MP Biomedicals) at 4 m s^−1^ for 20 s and centrifugation (1 min, 13 000 rpm). One microlitre of the supernatant was then used directly as the template for the PCR.

### Generation of the *S. cerevisiae* YEDIS‐G2 strain

A 3216‐bp DNA fragment was assembled *in vitro* by overlap extension PCR (Nelson and Fitch, 2011) from four independent fragments previously generated by PCR (Fig. S1): (i) the *GAL1* promoter obtained from plasmid PCM185 (Gari *et al*., 1997) with primers A‐GAL1 and B‐GAL1; (ii) the coding sequence for the α‐factor signal sequence obtained with primers C‐MATα and D‐MATα from the genome of *S. cerevisiae* BY4742 (*MATα*,* his3*,* leu2*,* lys2*,* ura3*) (Brachmann *et al*., 1998); (iii) the auxotrophic marker *URA3* obtained from plasmid pGREG506 (Jansen *et al*., 2005) with primers E‐URA3 and F‐URA3‐LEU2; and (iv) a truncated copy of the auxotrophic marker *LEU2* (containing the last two‐thirds of the CDS and the terminator region) obtained from pGREG505 (Jansen *et al*., 2005) with primers G‐LEU2 and H‐LEU2. Primer A‐GAL1 contains a 40 nt tail overlapping with the genomic region immediately preceding the *LEU2* deletion site in the *S. cerevisiae* strain BY4741 (*MATa*,* his3*,* leu2*,* met15*,* ura3*), while primer H‐LEU2 contains no tail because the end region of the truncated *LEU2* copy amplified with primers G‐LEU2 and H‐LEU2 is also present after the *LEU2* deletion site in BY4741 (Fig. S1). The rest of the primers contain tails homologous to adjacent fragments appropriate for overlap extension PCR (Table S2). Fusion of the four fragments was carried out in two steps: first, the *GAL1* promoter was fused with the coding sequence for the α‐factor signal sequence and the auxotrophic marker *URA3* was fused with the truncated copy of the auxotrophic marker *LEU2*; and second, the two resulting fragments were fused to one another. The resulting 3216 bp DNA fragment was then transformed into *S. cerevisiae* BY4741, Ura^+^ transformants were screened by PCR, and one of the positives was characterized by partial sequencing (Fig. S1) and shown to contain the correct DNA construct integrated into its genome at the *LEU2* deletion site. The new strain was named YEDIS‐G2.

### Synthesis of Fragment B

A synthetic DNA fragment (Integrated DNA Technologies, Belgium) with the coding region for the *c*‐*myc* and FLAG epitopes, a 6xHis purification tag and the *S. cerevisiae MET15* terminator was first cloned between the *Pvu*II sites of pBuescript KS+. The codons used in the synthetic fragment were chosen bearing in mind the codon bias for *P. pastoris* and *S. cerevisiae*, obtained from the Codon Usage Database (Nakamura *et al*., 2000). Then, a fragment of the *S. cerevisiae LEU2* gene comprising the promoter and the first two‐thirds of the CDS was amplified by PCR from pGREG505 (Jansen *et al*., 2005), with primers PC‐LEU2 and PD‐LEU2, and cloned at the *Bam*HI and *Hind*III sites located at the 3′ end of the synthetic fragment, generating the plasmid pBSFS+23LEU2 (Fig. S3).

Fragment B (1466 bp) was routinely obtained by PCR from plasmid pBSFS+23LEU2 with primers FragB‐FW and FragB‐RV (Fig. S3), and was used to drive the integration of the heterologous genes to be expressed into the genome of *S. cerevisiae* YEDIS‐G2, regenerating the auxotrophic marker *LEU2*, and to fuse the tags described above at the *C*‐terminus of the recombinant proteins (Fig. 1A).

### Construction of the pYEDIS vector

In first place, a 2849 bp DNA fragment from the plasmid pPICZα‐A (Life Technologies) was amplified with primers TP‐pPIC‐FW and TP‐pPIC‐RV to serve as backbone for the construction. This fragment contains the *P. pastoris AOX1* promoter, an *E. coli* origin of replication and the *Sh ble* gene for selection of *P. pastoris* and *E. coli* transformants on Zeocin‐containing plates (Fig. S4). To be fused to this plasmid backbone, four other DNA fragments were amplified by PCR: (i) a 312 bp fragment encoding the *S. cerevisiae* α‐factor signal sequence, amplified from genomic DNA of the *S. cerevisiae* YEDIS‐BcIEB1 strain (which derives from strain YEDIS‐G2 and expresses the BcIEB1 protein by insertion of the *Bcieb1* gene in the genome) with primers TP‐A‐FW and TP‐A‐RV; (ii) a 2261 bp fragment encoding the *c*‐*myc*, FLAG and 6xHIS tags, the *MET15* terminator and the *S. cerevisiae LEU2* gene, also from genomic DNA of *S. cerevisiae* YEDIS‐BcIEB1, with primers TP‐CL‐FW and TP‐CL‐RV; (iii) a 1388 bp fragment carrying the yeast 2 micron origin of replication, amplified from plasmid pNDN‐OGG (Schumacher, 2012) with primers TP‐2μ‐FW and TP‐2μ‐RV; and (iv) a 558 bp fragment containing the *S. cerevisiae GAL1* promoter, from plasmid pGREG506 with primers TP‐G‐FW and TP‐G‐RV. Each primer pair included tails to generate ends overlapping with the next adjacent region (Fig. S4), to allow fusion by the Gibson assembly method (Gibson *et al*., 2009). The four latter fragments were fused with the Gibson Assembly Cloning Kit (New England Biolabs) and re‐amplified with primers TP‐G‐FW and TP‐2μ‐RV to obtain the final product of 4398 bp. Finally, this DNA construct was fused to the plasmid backbone described above and transformed into *E. coli* to obtain the pYEDIS vector (Fig. S4). One *Pvu*II restriction site and two *Not*I restriction sites were introduced during the assembly process to be used for the expression of recombinant proteins in *S. cerevisiae* and *P. pastoris* as explained above.

Partial sequencing of the pYEDIS plasmid resulted in the expected sequence, except for the deletion of one of five adenines spanning codons 2 and 3 of the *c*‐*myc* epitope, what results in the appearance of a stop codon (Fig. S4C). This alteration does not affect the use of pYEDIS for protein expression, since homologous recombination with the gene to be expressed (Fragment A in Fig. 1B) restores the correct sequence. On the contrary, this new and unexpected stop codon in the pYEDIS sequence was advantageous, since any yeast cell transformed by chance with the self‐recirculated or undigested vector, that is, without Fragment A, will not be detected by anti‐*c‐myc* antibodies in the colony blot screening, thus reducing the appearance of false positives.

### Generation of the *P. pastoris* YEDIS strain

The *P. pastoris* necessary as a recipient for the expression of the pYEDIS+GOI plasmids obtained from *S. cerevisiae* (Fig. 1C) was obtained by integration, into the *AOX1* promoter of *P. pastoris* GS115, of a linear DNA fragment generated as follows. The pYEDIS vector was digested with *Not*I and re‐ligated to remove the *GAL1* promoter. The re‐ligated vector was then used as template for the amplification, with primers AOX‐FW and MATα‐RV, of a 1221 bp DNA fragment carrying the *AOX1* promoter fused with the coding sequence for the *S. cerevisiae* α‐factor signal peptide, which ends in a stop codon introduced with the MATα‐RV primer (Fig. S5). On the other hand, a second 2731 bp fragment containing the *P. pastoris HIS4* gene was amplified from the genome of *P. pastoris* X33, using primers HIS4‐FW and HIS4‐RV, which introduced ends overlapping with the previous fragment. The two PCR products were fused by their two ends with the Gibson Assembly Cloning Kit, generating a circular 3904 bp DNA molecule (Fig. S5). This circular DNA fragment was then used as template in a third PCR, with primers PAOXmid‐FW and PAOXmid‐RV, amplifying the whole circular molecule from the middle of the *AOX1* promoter (Fig. S5). Five micrograms of the purified amplicon was then electroporated into *P. pastoris* GS115 (*his4* ^−^), and the transformants were selected on minimal medium without histidine. Positive transformants were identified by colony PCR and characterized by partial sequencing (Fig. S5). The resulting strain was named *P. pastoris* YEDIS.

## Conflict of interest

The authors have no conflict of interests associated with this research work.

## Supporting information


**Table S1.** Initial set of *B. cinerea* genes selected for its expression in *S. cerevisiae* and *P. pastoris* in order to test the YEDIS system.
**Table S2.** Oligonucleotides used in this work.
**Table S3.** *B. cinerea* proteins selected for high throughput expression experiments using the pYEDIS vector.
**Fig. S1.** Construction and genomic characterization of the *S. cerevisiae* YEDIS‐G2 strain. (A) Transforming DNA was assembled in vitro by Overlap Extension PCR from four independent PCR products (Fragments 1‐4), which had been previously amplified with the indicated primers. The ends of the transforming DNA had homology (introduced with primers *A‐GAL1* and *H‐LEU2*) with the borders of the scar left by the deletion of *LEU2* in strain BY4741. (B) Three independent PCRs were carried out to analyse the correct integration of the transforming DNA in 20 of the Ura+ transformants. PCR products obtained for two positive transformants (YEDIS‐G2.1 and YEDIS‐G2.2) or the parental strain (BY4741), using the indicated primers, are shown as an example. (C) The relevant region in the genome of the strain YEDIS‐G2.1 was partially sequenced (light blue arrows) using as templates the PCR products showed in (B) and the indicated primers.
**Fig. S2.** Design of primers for the expression of secretory proteins with the YEDIS system. In order to express the whole mature amino acid sequence coded by the GOI, the forward primer should bind right after the region coding for the GOI's own signal sequence in the cDNA, and the reverse primer should bind right before the stop codon. Both primers should have the indicated tails to allow the required recombination in the yeast cells. Although 40‐nt tails are shown, the use of shorter ones is also possible although with lower recombination efficiency (see text for details).
**Fig. S3.** Map and partial sequence of plasmid pBSFS+23LEU2. (A) Map of the plasmid showing the relevant features and the primers binding sites (red arrows). CSF: chemically‐synthesized fragment that comprises a region coding for the c‐*myc* and FLAG epitopes, the 6xHis purification tag, and the *S. cerevisiae MET15* terminator. AMPR: Ampicillin resistance gene. Ori: pBR322 origin of replication. The region labelled as 3’‐truncated *LEU2* carries the *LEU2* promoter and the first two thirds of the *LEU2* CDS. (B) Partial sequence of pBSFS+23LEU2, showing the FragB‐FW and FragB‐RV binding sites and other relevant elements. Note that part of the 3’‐truncated *LEU2* region has been omitted for clarity (…).
**Fig. S4.** Map and features of the pYEDIS vector. (A) Vector map. The location of all primers used to generate the five PCR fragments that were assembled *in vitro* to generate the pYEDIS plasmid (see Material and Methods) are shown as red arrows. Note the location of the single *Pvu*II restriction site used to linearize the plasmid and introduce the GOI, as well as the two *Not*I restriction sites used to remove the *GAL1* promoter prior to transformation of *P. pastoris* YEDIS. Semi‐circular light‐blue arrows correspond to the regions of the plasmid that were sequenced, and displayed the expected sequence with the exception of the indicated new stop codon (STOP*) discussed in the text. (B) Location, length and description of all the elements in the pYEDIS vector. (C) Direct sequencing of plasmid pYEDIS revealed that the expected five consecutives adenines in codons 1 and 2 of c‐*myc* (in red) were actually four adenines, changing the reading frame and causing the appearance of the new stop codon (STOP* in panel (A)).
**Fig. S5.** Generation of *P. pastoris* YEDIS. (A) A DNA fragment containing the *P. pastoris AOX1* promoter (P*AOX1*) and the α factor signal sequence (MATα), amplified with primers AOX‐FW and MATα‐RV, was fused *in vitro* by its two ends with a second PCR product carrying the *P. pastoris HIS4* gene, amplified with primers HIS4‐FW and HIS4‐RV. Fusion was possible by the ends introduced by the primers HIS4‐FW and HIS4‐RV, homologous to the α factor signal sequence and the *HIS4* gene respectively. The resulting circular molecule was then amplified with primers PAOXmid‐FW and PAOXmid‐RV, resulting in the depicted linear DNA fragment used to transform *P. pastoris* GS115. Integration of the transforming DNA into P*AOX1* results in the *P. pastoris* YEDIS strain. (B) Three PCR reactions were carried out to analyse the correct genomic integration of the transforming DNA in the transformants. PCR products obtained for two positive transformants (YEDIS.1 and YEDIS.2) or the parental strain (GS115), using the indicated primers, are shown. (C) Scheme of the *P. pastoris* YEDIS genome, showing the *Not*I restriction site designed to be the site of integration for the pYEDIS plasmid (see text for details). The PCR products described in (B) were partially sequenced as indicated by light blue arrows marked with the primers used in the sequencing reactions.
**Fig. S6.** Effect of the length of recombination tails on the frequency of transformants expressing the *B. cinerea* protein BcIEB1. (A) Effect of the length of the tails on the number of transformants obtained. *S. cerevisiae* YEDIS‐G2 was co‐transformed with the *Bcieb1* gene, flanked by recombination tails for the indicated length, and with the Fragment B. In the control, the *Bcieb1* gene was omitted from the transformation mix, but the Fragment B was maintained. (B) Colony blot screening of transformants for BcIEB1 expression. Thirteen randomly chosen colonies from each transformation were screened by colony blot with anti‐c‐*myc* antibodies as explained in the legend to Figure 2a.
**Fig. S7.** High‐throughput expression of *B. cinerea* proteins in *S. cerevisiae* using the pYEDIS vector. (A) PCR amplification of 23 *B. cinerea* genes (see details in table S3) with primers including 30‐nt recombination tails (Table S2) and using 50 ng of *B. cinerea* cDNA as template. (B) Colony PCR with primers MATα‐check‐FW y M15‐LEU‐check‐RV of 2‐4 colonies obtained by co‐transformation of *S. cerevisiae* BY4741 with *Pvu*II‐digested pYEDIS vector and the amplicons shown in (A). The scheme indicates primer binding sites.
