# Supplementary material for: New tools for high‐throughput expression of fungal secretory proteins in Saccharomyces cerevisiae and Pichia pastoris
Source: Microb Biotechnol. 2018 Oct 5;12(6):1139–53. doi: 10.1111/1751-7915.13322 (PMC6801181; doi:10.1111/1751-7915.13322)
Supplement: Supplementary file 1 — Table S1. Initial set of B. cinerea genes selected for its expression in S. cerevisiae and P. pastoris in order to test the YEDIS system. Table S2. Oligonucleotides used in this work. Table S3. B. cinerea proteins selected for high throughput expression experiments using the pYEDIS vector. Fig. S1. Construction and genomic characterization of the S. cerevisiae YEDIS‐G2 strain. (A) Transforming DNA was assembled in vitro by Overlap Extension PCR from four independent PCR products (Fragments 1‐4), which had been previously amplified with the indicated primers. The ends of the transforming DNA had homology (introduced with primers A‐GAL1 and H‐LEU2) with the borders of the scar left by the deletion of LEU2 in strain BY4741. (B) Three independent PCRs were carried out to analyse the correct integration of the transforming DNA in 20 of the Ura+ transformants. PCR products obtained for two positive transformants (YEDIS‐G2.1 and YEDIS‐G2.2) or the parental strain (BY4741), using the indicated primers, are shown as an example. (C) The relevant region in the genome of the strain YEDIS‐G2.1 was partially sequenced (light blue arrows) using as templates the PCR products showed in (B) and the indicated primers. Fig. S2. Design of primers for the expression of secretory proteins with the YEDIS system. In order to express the whole mature amino acid sequence coded by the GOI, the forward primer should bind right after the region coding for the GOI's own signal sequence in the cDNA, and the reverse primer should bind right before the stop codon. Both primers should have the indicated tails to allow the required recombination in the yeast cells. Although 40‐nt tails are shown, the use of shorter ones is also possible although with lower recombination efficiency (see text for details). Fig. S3. Map and partial sequence of plasmid pBSFS+23LEU2. (A) Map of the plasmid showing the relevant features and the primers binding sites (red arrows). CSF: chemically‐synthesized fragment that co [file MBT2-12-1139-s001.pdf]

Table S1. Initial set of *B. cinerea* genes selected for its expression in *S. cerevisiae* and *P. pastoris* in order to test the YEDIS system

Protein	Genome ID[a]	Predicted signal peptide sequence[b]	Predicted MW[c] (Da)
BcSPL1	Bcin03g00500	MQFPTLATLLTFAVSATA	15 278
BcIEB1	Bcin15g00100	MFSKTFIATLLASSAAA	20 501
BcSUN1	Bcin06g06040	MKFTPVSVALLSVAGVAIA	49 290
BcXyn11A	Bcin03g00480	MVSASSLLLAASAIAGVFS	25 189
BcXyn11B	Bcin15g01600	MITFSSLLVTFSAISTSLA	25 003
BcXyn11C	Bcin12g00090	MVSYKAFLITLAAVTRVLT	30 569

[a]ID of the gene in the *B. cinerea* B05.10 genome database (http://fungi.ensembl.org/Botrytis_cinerea).
[b]Amino acid sequence of the signal peptide predicted by the SignalP 4.1 server (www.cbs.dtu.dk/services/SignalP).
[c]Theoretical molecular weight calculated for the mature recombinant protein sequence (including the epitopes and purification tag, but excluding the α-factor signal sequence).

Table S2. Oligonucleotides used in this work

Primer ID	Primer sequence (5' → 3')<br>(In the case of primers for the amplification of the genes to be expressed in yeast, tails designed for homologous recombination are in lower case)	Details
IEB1-FW	agaagaaggggtatctttggataaaagagaggctgaagctACCCCCATTGTCTCTGCAC	Amplification of the *Bcieb1* gene for expression with the YEDIS system, each primer includes a 40-nt tail.
IEB1-RV	aatcaccacccaaatcttcttcagaaatcaatttttgttcAGCGTACTCCCAAGCGGA	
IEB1_30_FW	gtatctttggataaaagagaggctgaagctACCCCCATTGTCTCTGCAC	Amplification of the *Bcieb1* gene to test the homologous recombination efficiency of 30-nt tails.
IEB1_30_RV	caaatcttcttcagaaatcaatttttgttcAGCGTACTCCCAAGCGGA	
IEB1_20_FW	ataaaagagaggctgaagctACCCCCATTGTCTCTGCAC	Amplification of the *Bcieb1* gene to test the homologous recombination efficiency of 20-nt tails.
IEB1_20_RV	tcagaaatcaatttttgttcAGCGTACTCCCAAGCGGA	
SUN1-FW	agaagaaggggtatctttggataaaagagaggctgaagctCAACCCCACAACCATCAACAC	Amplification of the *Bcieb1* gene for expression with the YEDIS system, each primer includes a 40-nt tail.
SUN1-RV	aatcaccacccaaatcttcttcagaaatcaatttttgttcTGATGAGAAGACGTATGTAGCGG	
SPL1-FW	agaagaaggggtatctttggataaaagagaggctgaagctATCACCGTCTCCTACGACGTTGG	Amplification of the *BcSpl1* gene for expression with the YEDIS system, each primer includes a 40-nt tail.
SPL1-RV	aatcaccacccaaatcttcttcagaaatcaatttttgttcCAATCCACAAGCACTCTTGTCGAC	
XYN11A_FW	gtatctttggataaaagagaggctgaagctCCAGCCGCGGCACCC	Amplification of the *BcXyn11A* gene for expression with the YEDIS system, each primer includes a 30-nt tail.
XYN11A_RV	caaatcttcttcagaaatcaatttttgttcAGAAACAGTGATGGAAGCGG	
XYN11B_FW	gtatctttggataaaagagaggctgaagctATTCCGGGTTCCATTCTCC	Amplification of the *BcXyn11B* gene for expression with the YEDIS system, each primer includes a 30-nt tail.
XYN11B_RV	caaatcttcttcagaaatcaatttttgttcACTAACCGTCTGCGTAGCAGC	
XYN11C_FW	gtatctttggataaaagagaggctgaagctTATCCAGCCAACTCAAGTTTTG	Amplification of the *BcXyn11C* gene for expression with the YEDIS system, each primer includes a 30-nt tail.
XYN11C_RV	caaatcttcagaaatcaatttttgttcCAAGCATTGAGAATAGTAAGAATTTGC	
5'AOX	GACTGGTTCCAATTGACAAGC	Amplification of insert (GOI) from pYEDIS+GOI
MATα-check-FW	CCAACAGCACAAATAACGG	
M15-LEU-check-RV	ATGAACAGGATCCACTTGTG	
TP-2u-FW	GTGAAAGTTCCCTCAAGAATGTATGATCCAATATCAAAGGAAATG	Generation of the pYEDIS vector
TP-2u-RV	TTGAAGCTATGGTGTGTGGGTGAACGAAGCATCTGTGC	
TP-A-FW	ATTCTAGAGCGGCCGCAAAAATGAGATTTCCTTCAATTTTTACTGC	Generation of the pYEDIS vector
TP-A-RV	TCAGAAATCAATTTTTGTTCCAGCTGAGCTTCAGCCTCTCTTTTATCC	

TP-CL-FW	ATAAAAGAGAGGCTGAAGCTCAGCTGGAACAAAAATTGATTTCTGAAGAAG	Generation of the pYEDIS vector
TP-CL-RV	CCTTTGATATTGGATCATACATTCTTGAGGGAACTTTCACC	
TP-G-FW	AACAACTAATTATTCGAAACGCGGCCGCGAACAAAAGCTGGAGCTCG	Generation of the pYEDIS vector
TP-G-RV	AATTGAAGGAAATCTCATTTTTGCGGCCGCTCTAGAATCC	
TP-pPIC-FW	AAGCACAGATGCTTCGTTCACCCACACACCATAGCTTCAAAATG	Generation of the pYEDIS vector
TP-pPIC-RV	CGAGCTCCAGCTTTTGTTCGCGGCCGCGTTTCGAATAATTAGTTGTTTTTTG	
AOX-FW	AGATCTAACATCCAAAGACGAAAG	Amplification of a 1221-bp DNA fragment, carrying the *AOX1* promoter and the *S. cerevisiae* α-factor, which was used for the construction of the quimeric DNA sequence necessary for the generation of *P. pastoris* YEDIS strain.
MATα-RV	TTAAGCTTCAGCCTCTCTTTTATC	
HIS4-FW	GATAAAAGAGAGGCTGAAGCTTAAGAAACACTGGATGTTAGATGCC	Amplification of the *P. pastoris HIS4* marker, which was used for the construction of the quimeric DNA sequence necessary for the generation of the *P. pastoris* YEDIS strain.
HIS4-RV	CTTTCGTCTTTGGATGTTAGATCTATCTCCTGATGACTGACTCACTG	
PAOXmid-FW	CTCCAGATGAGGGCTTTCTGAG	Amplification of a 3904-bp DNA fragment, necessary for the generation of *P. pastoris* YEDIS strain.
PAOXmid-RV	TGATGTTCGGGTGTAATGCG	
A-GAL1	GATCCATGTATAATCTTCATTATTACAGCCCTCTTGACCTGAACAAAAGCTGGAGCTCG	Amplification of *GAL1* promoter from PCM185 plasmid, to generate the *S. cerevisiae* YEDIS-G2 strain.
B-GAL1	AAAATTGAAGGAAATCTCATACTAGTGCGGCCGCTCTAG	
C-MATα	TCTAGAGCGGCCGCACTAGTATGAGATTTCCTTCAATTTTTACTG	Amplification of the α-factor signal sequence from the genome of *S. cerevisiae* BY4742, to generate the *S. cerevisiae* YEDIS-G2 strain.
D-MATα	ATTGAAAAGCTGTGGTATGGTCAAGCTTCAGCCTCTCTTTTATC	
E-URA3	AAAAGAGAGGCTGAAGCTTGACCATACCACAGCTTTTCAATTC	Amplification of *S. cerevisiae URA3* from plasmid PGREG506, to generate the *S. cerevisiae* YEDIS-G2 strain.
F-URA3-LEU2	GTACAATTGAAGTTCTTTACGCATCTGTGCGGTATTTCAC	
G-LEU2	GTGAAATACCGCACAGATGCGTAAAGAACTTCAATTGTACGCC	Amplification of a truncated copy of *LEU2* (last two thirds of CDS + terminator) from pGREG505, to generate the *S. cerevisiae* YEDIS-G2 strain.
H-LEU2	ATTCTTGAGGGAACTTTCACC	
FragB-FW	GAACAAAAATTGATTTCTGAAGAAG	Amplification of Fragment B from plasmid pBSFS+23LEU2
FragB-RV	AACCTGGGATAACGGAGG	
PC-LEU2	CGCGGATCCTGTTCATGTGTGTTCAAAAACG	Amplification of a DNA fragment comprising the promoter and the first two thirds of CDS of *LEU2*, from pGREG505 plasmid
PD-LEU2	CCCAAGCTTGCAACAAACCCAAGGAACC	
Yedis8_FW	gtatctttggataaaagagaggctgaagctACACCAATTGCCCAAGTCAAG	High throughput expression in *S. cerevisiae* of the genes indicated in Table S3.
Yedis8_RV	caaatcttcttcagaaatcaatttttgttcTGCGGCAGTAGCGAAACC	

Yedis9_FW	gtatctttggataaaagagaggctgaagctGCTCCAGCACCAGCACC	
Yedis9_RV	caaatcttcttcagaaatcaatttttgttcACACTTGACACCAGATGGGAG	
Yedis10_FW	gtatctttggataaaagagaggctgaagctAGCCCTGCTTTAGTAAACCGC	
Yedis10_RV	caaatcttcttcagaaatcaatttttgttcCATGTAAGAAGCATCGTAGTAGAACTCG	
Yedis11_FW	gtatctttggataaaagagaggctgaagctATGGCCACTGGTATCACAGTAAC	
Yedis11_RV	caaatcttcttcagaaatcaatttttgttcATAGATGTTGATAGTTTGGCCTGG	
Yedis12_FW	gtatctttggataaaagagaggctgaagctGCTCCAGGTTCCGCCC	
Yedis12_RV	caaatcttcttcagaaatcaatttttgttcGCAAGAAGCTCCGGTAGGAAC	
Yedis13_FW	gtatctttggataaaagagaggctgaagctGTTAACCAAGGTTTCAACTATGGTG	
Yedis13_RV	caaatcttcttcagaaatcaatttttgttcCAAAGCAGCTGCAACGG	
Yedis14_FW	gtatctttggataaaagagaggctgaagctTTGCCCACTTTCGGCAA	
Yedis14_RV	caaatcttcttcagaaatcaatttttgttcAGTAGAATTACTGATTGCCAACAACTTAG	
Yedis15_FW	gtatctttggataaaagagaggctgaagctATTTCACCGAGAGGTTCGG	
Yedis15_RV	caaatcttcttcagaaatcaatttttgttcACACCCTCCACTACTTCCTCC	
Yedis16_FW	gtatctttggataaaagagaggctgaagctCAGAGTCAAGGTTATGCTCAATG	
Yedis16_RV	caaatcttcttcagaaatcaatttttgttcCAATGCTGCCATGATTGC	
Yedis17_FW	gtatctttggataaaagagaggctgaagctGCTCCAACAATCGAGTCGAG	
Yedis17_RV	caaatcttcttcagaaatcaatttttgttcAATCCAAACCGGAAACAACA	
Yedis18_FW	gtatctttggataaaagagaggctgaagctGCTCCATCTCCAGTTGAGAATC	
Yedis18_RV	caaatcttcttcagaaatcaatttttgttcCAAAAGTGCAGCACCAACA	
Yedis19_FW	gtatctttggataaaagagaggctgaagctGAGTCCTCCCTGGAGCTCTT	
Yedis19_RV	caaatcttcttcagaaatcaatttttgttcATCTTTCTTATTAACCAACTTCAACAAAG	
Yedis20_FW	gtatctttggataaaagagaggctgaagctGCCCCAGGTACTGCTTTG	
Yedis20_RV	caaatcttcttcagaaatcaatttttgttcAACGTATTTGCAAGTAACACCAG	
Yedis21_FW	gtatctttggataaaagagaggctgaagctCTCTCGGCCGCAAGCT	
Yedis21_RV	caaatcttcttcagaaatcaatttttgttcCAACCCACAAACCCCACTC	
Yedis22_FW	gtatctttggataaaagagaggctgaagctGAGCACCCATTTCTTCGC	
Yedis22_RV	caaatcttcttcagaaatcaatttttgttcATATCTTCGTCTATGTCCTCCTCC	
Yedis23_FW	gtatctttggataaaagagaggctgaagctCAAAGTGATTCATACGTCAATCAAAC	

Yedis23_RV	caaatcttcttcagaaatcaatttttgttcAACCACCGGGATCTTCACC	
Yedis24_FW	gtatctttggataaaagagaggctgaagctGCTCCCCAGAATATTCAGTATCAC	
Yedis24_RV	caaatcttcttcagaaatcaatttttgttcAGTCCAAGCTAAAGGATCCGG	
Yedis25_FW	gtatctttggataaaagagaggctgaagctCTCCCACAATCTCCTACAGACAC	
Yedis25_RV	caaatcttcttcagaaatcaatttttgttcAGGAACCTCGAAATAACTAAAGTAATCC	
Yedis26_FW	gtatctttggataaaagagaggctgaagctTGGTCTCCATTCTCTTTCCTTTAC	
Yedis26_RV	caaatcttcttcagaaatcaatttttgttcAGGTCTCTCAACTGCAAGGTCA	
Yedis27_FW	gtatctttggataaaagagaggctgaagctCAAACATTCACTGATTGCAACC	
Yedis27_RV	caaatcttcttcagaaatcaatttttgttcCATAACGAGATAACCTAATCCGAGAC	
Yedis28_FW	gtatctttggataaaagagaggctgaagctGCGGATCTCCCCCCTATC	
Yedis28_RV	caaatcttcttcagaaatcaatttttgttcCAACATGATCATACCCGCAC	
Yedis29_FW	gtatctttggataaaagagaggctgaagctCAGCTTGATACGCTTGCCA	
Yedis29_RV	caaatcttcttcagaaatcaatttttgttcTGCCGGAATGCACTGC	
Yedis30_FW	gtatctttggataaaagagaggctgaagctATTGCCACAATCTCTGTAACCG	
Yedis30_RV	caaatcttcttcagaaatcaatttttgttcTAGCCAGTACATGAAACCGAACA	

Table S3. *B. cinerea* proteins selected for high throughput expression experiments using the pYEDIS vector

Name used in this work	Protein name and reference(s), when available	Genome ID[a]	Predicted signal sequence[b]	MW[c] (Da)
Yedis 4	BcXyn11A (Brito, Espino, & González, 2006)	Bcin03g00480	MVSASSLLLAASAI AGVFS	25 189
Yedis 8	BcAP8 (Espino et al., 2010; ten Have et al., 2010)	Bcin12g02040	MLFTSLLALAGAA AVTA	41 34
Yedis 9	BcPG1 (ten Have, Mulder, Visser, & van Kan, 1998)	Bcin14g00850	MVQLLSMASGLLA LSAIVS	39 192
Yedis 10	BcPME2 (Kars, McCalman, Wagemakers, & van Kan, 2005)	Bcin03g03830	MRSFALLSLTASLL GSVSA	39 296
Yedis 11	BcEkDa (van der Vlugt-Bergmans, Wagemakers, & van Kan, 1997)	Bcin15g00520		11 896
Yedis 12	BcPG2 (Kars et al., 2005)	Bcin14g00610	MVHITSLISFLASTA LVSA	38 969
Yedis 13		Bcin09g00200	MLSKTLLAVAASLS TASA	45 62
Yedis 14		Bcin15g03150	MLLLNIFSVAFAAS AVQA	63 125
Yedis 15		Bcin12g03390	MLFSRTATILSLLCV QATA	68 164
Yedis16	Xyn10A (García, González, González, & Brito, 2017)	Bcin03g03480	MFSALNLATLAAM LSHINLALA	41 889
Yedis 17		Bcin06g06670	MVSIRHLAIAASSIL SFVSA	22 31
Yedis 18		Bcin14g03970	MKTFHTIATLAAAG SALA	49 144
Yedis 19		Bcin09g01190	MKFSRVVSGLAVC SFAAC	67 603
Yedis 20		Bcin15g02380	MKFSTVAATAVLA GSAVA	27 835
Yedis 21		Bcin02g01420	MRPIIFLQHLLLISL NLSPITA	55 041
Yedis 22		Bcin08g02390	MKWSIVSAISLLAA SAAA	61 663
Yedis 23		Bcin14g00810	MSLRKFLPVASLFG LATA	56 599
Yedis 24		Bcin03g05600	MFGLEVITIAALSG VALA	50 337
Yedis 25		Bcin07g02940	MIFPSIILALFTLSTA	39 069

Yedis 26		Bcin05g04010	MQLPAVLLALSLLP GALS	22 349
Yedis 27		Bcin01g06010	MRSSTISASAVVLL SGLASA	41 675
Yedis 28		Bcin02g06940	MKTFGFASAAAAA LTGALFASSAAA	57 514
Yedis 29	Xyn10B (García et al., 17)	Bcin05g06020	MHISNLITLAILPLA YG	52 665
Yedis 30		Bcin05g05090	MFSKSVARAGLIAS LAITTVNA	46 769

[a]ID of the gene in *B. cinerea* B05.10 genome database (http://fungi.ensembl.org/Botrytis_cinerea)

[b]Amino acid sequence of the signal peptide predicted by the SignalP 4.1 server (www.cbs.dtu.dk/services/SignalP).

[c]Theoretical molecular weight calculated for the mature recombinant protein sequence (including the epitopes and purification tag, but excluding the α-factor signal sequence).

References in Table S3:

Brito, N., Espino, J. J., and González, C. (2006) The endo-ß-1,4-xylanase Xyn11A is required for virulence in *Botrytis cinerea*. *Mol. Plant Microbe Interact.* **19**: 25-32.

Espino, J. J., Gutiérrez-Sánchez, G., Brito, N., Shah, P., Orlando, R., and González, C. (2010) The *Botrytis cinerea* early secretome. *Proteomics* **10**: 3020-3034.

García, N., González, M. A., González, C., and Brito, N. (2017) Simultaneous silencing of xylanase genes in *Botrytis cinerea*. *Front. Plant Sci.* **8**: 2174.

Kars, I., Krooshof, G. H., Wagemakers, L., Joosten, R., Benen, J. A. E., and van Kan, J. A. L. (2005a) Necrotizing activity of five *Botrytis cinerea* endopolygalacturonases produced in *Pichia pastoris*. *Plant J.* **43**: 213-225.

Kars, I., McCalman, M., Wagemakers, L., and van Kan, J. A. L. (2005b) Functional analysis of *Botrytis cinerea* pectin methylesterase genes by PCR-based targeted mutagenesis: *Bcpme1* and *Bcpme2* are dispensable for virulence of strain B05.10. *Mol. Plant Pathol.* **6**: 641-652.

ten Have, A., Mulder, W., Visser, J., and van Kan, J. A. (1998) The endopolygalacturonase gene *Bcpg1* is required for full virulence of *Botrytis cinerea*. *Mol. Plant Microbe Interact.* **11**: 1009-1016.

ten Have, A., Espino, J. J., Dekkers, E., Sluyter, S. C. V., Brito, N., Kay, J., González, C., and van Kan, J. A. (2010) The *Botrytis cinerea* aspartic proteinase family. *Fungal Genet. Biol.* **47**: 53-65.

van der Vlugt-Bergmans, C. J. B., Wagemakers, C. A. M., and van Kan, J. A. L. (1997) Cloning and expression of the cutinase A gene of *Botrytis cinerea*. *Mol. Plant Microbe Interact.* **10**: 21-29.

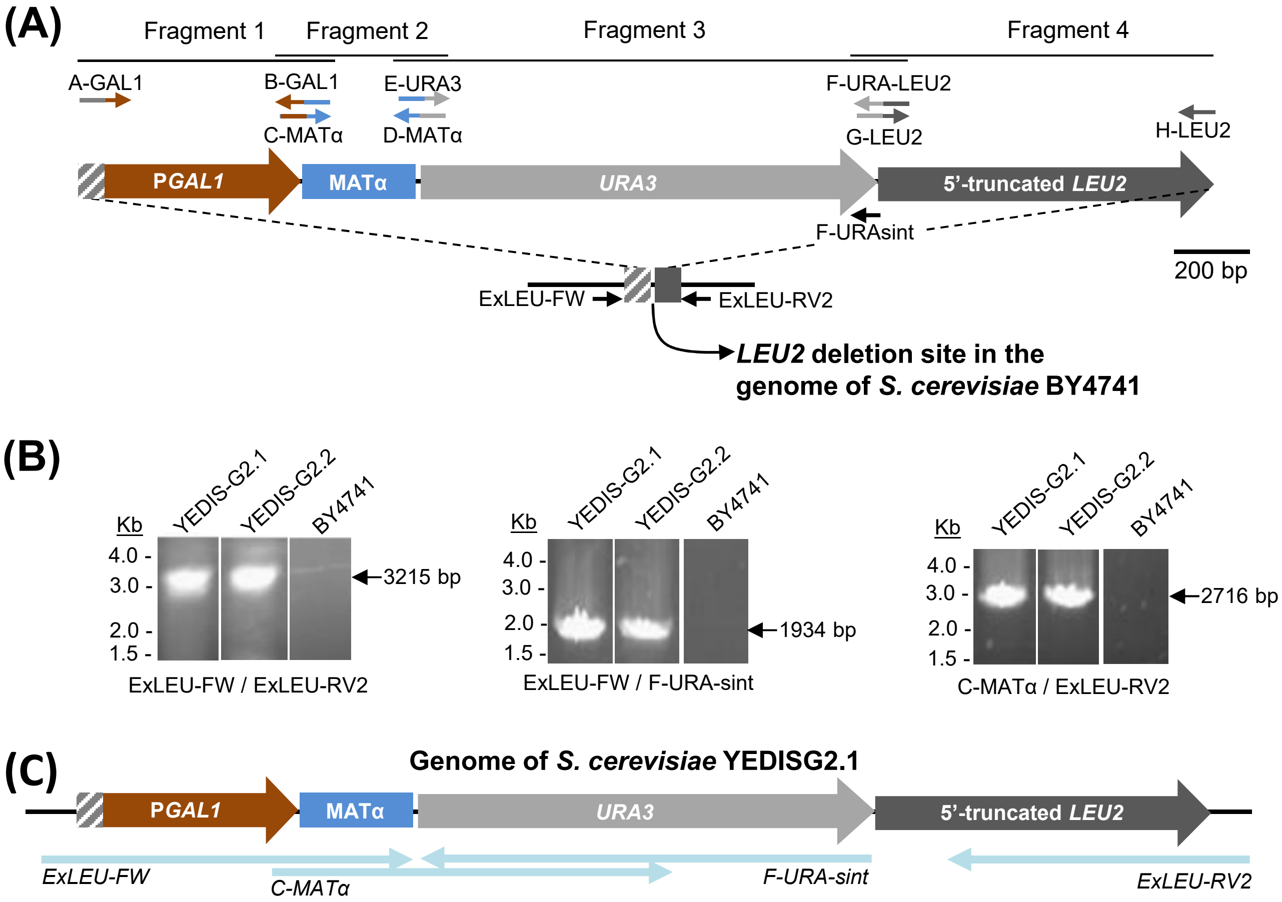

Fig. S1. Construction and genomic characterization of the *S. cerevisiae* YEDIS-G2 strain. (A) Transforming DNA was assembled in vitro by Overlap Extension PCR from four independent PCR products (Fragments 1-4), which had been previously amplified with the indicated primers. The ends of the transforming DNA had homology (introduced with primers *A-GAL1* and *H-LEU2*) with the borders of the scar left by the deletion of *LEU2* in strain BY4741. (B) Three independent PCRs were carried out to analyse the correct integration of the transforming DNA in 20 of the Ura^{+} transformants. PCR products obtained for two positive transformants (YEDIS-G2.1 and YEDIS-G2.2) or the parental strain (BY4741), using the indicated primers, are shown as an example. (C) The relevant region in the genome of the strain YEDIS-G2.1 was partially sequenced (light blue arrows) using as templates the PCR products showed in (B) and the indicated primers.

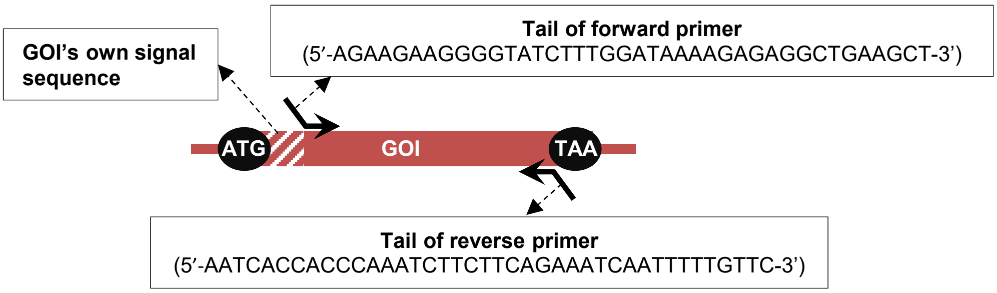

Fig. S2. Design of primers for the expression of secretory proteins with the YEDIS system. In order to express the whole mature amino acid sequence coded by the GOI, the forward primer should bind right after the region coding for the GOI's own signal sequence in the cDNA, and the reverse primer should bind right before the stop codon. Both primers should have the indicated tails to allow the required recombination in the yeast cells. Although 40-nt tails are shown, the use of shorter ones is also possible although with lower recombination efficiency (see text for details).

(A)

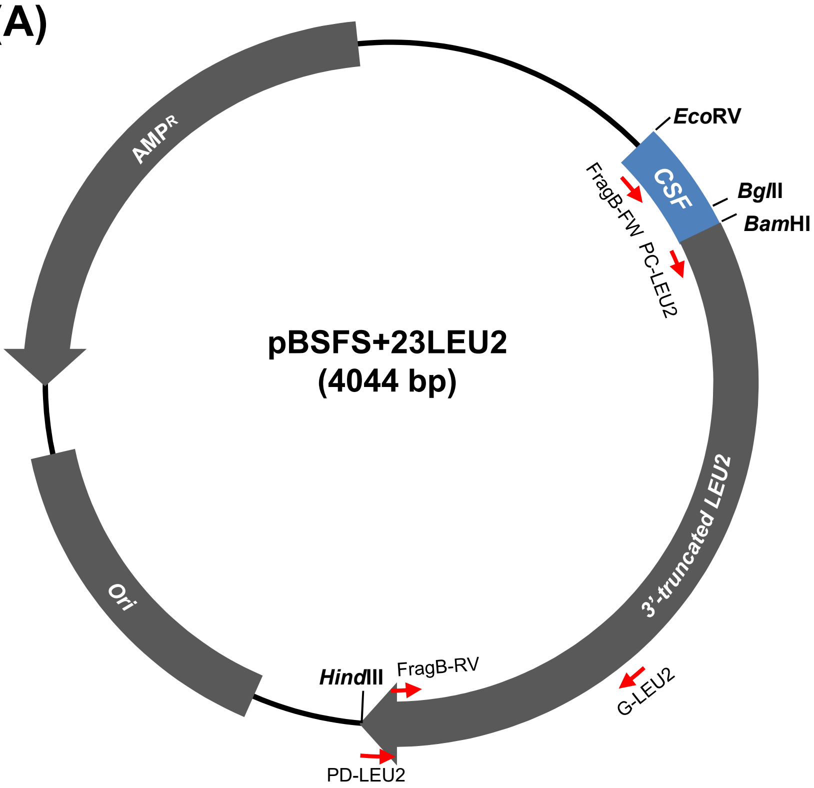

(B)

```
EcoRV                                                 FragB-FW
GATATC AGTTATTACCCTATGCGGTGTGAAATACCGCACAGATGC GAA CAA AAA TTG ATT TCT GAA GAA GAT TTG
                                                E   Q   K   L   I   S   E   E   D   L
                                                               c-myc

                                                                                  BglII
GGT GGT GAT TAT AAA GAT GAT GAT GAT AAA GGT GGT CAC CAT CAC CAT CAC CAT TAA AGATCT
 G   G   D   Y   K   D   D   D   D   K   G   G   H   H   H   H   H   H  Stop
                     FLAG                                6xHis

                                                                 BamHI
GTGTGCGTAATGAGTTGTAAAATTATGTATAAACCTACTTTCTCTCACAAGT GGATCC TGTTCATGTGTGTTCAAAAACG ...
                          TMET15                                   3'-truncated LEU2

                                  FragB-RV                                     HindIII
... GTG ATA TCA TCT CCG ATG AAG CCT CCG TTA TCC CAG GTT CCT TGG GTT TGT TGC AAGCTT
...      G   G   D   Y   K   D   D   D   D   K   G   G   S   L   G   L   L
                              3'-truncated LEU2
```

Fig. S3. Map and partial sequence of plasmid pBSFS+23LEU2. (A) Map of the plasmid showing the relevant features and the primers binding sites (red arrows). CSF: chemically-synthesized fragment that comprises a region coding for the c-*myc* and FLAG epitopes, the 6xHis purification tag, and the *S. cerevisiae MET15* terminator. AMP^{R}: Ampicillin resistance gene. Ori: pBR322 origin of replication. The region labelled as 3'-truncated *LEU2* carries the *LEU2* promoter and the first two thirds of the *LEU2* CDS. (B) Partial sequence of pBSFS+23LEU2, showing the FragB-FW and FragB-RV binding sites and other relevant elements. Note that part of the 3'-truncated *LEU2* region has been omitted for clarity (…).

(A)

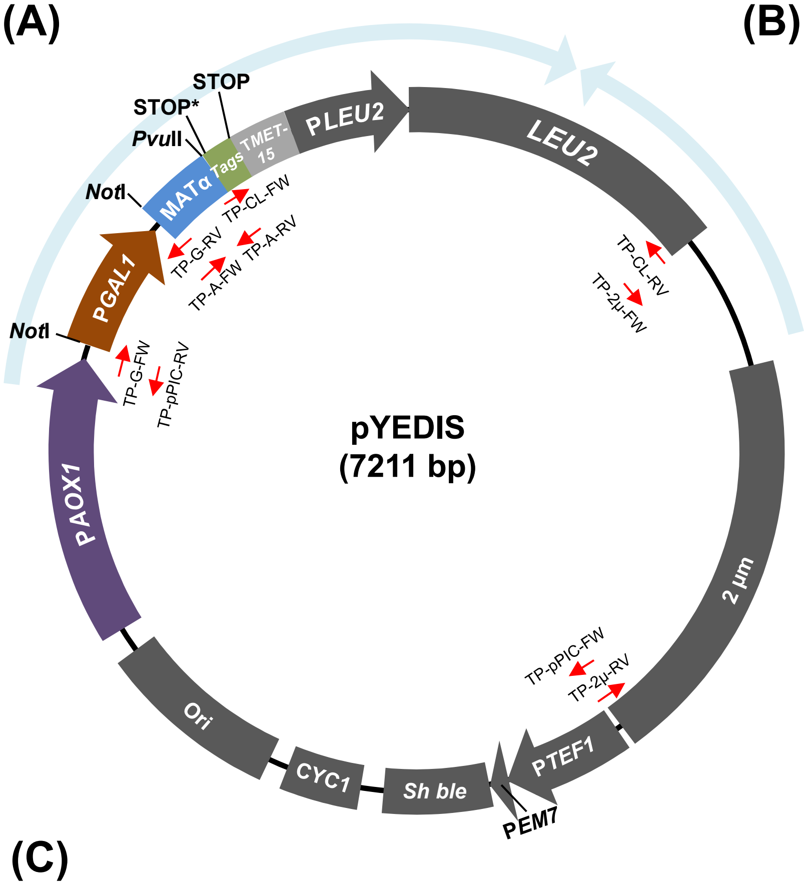

(B)

Name	Start (bp)	Lenght (bp)	Details
PAOX1	1	940	*P. pastoris AOX1* promoter
PGAL1	987	442	*S. cerevisiae GAL1* promoter
MATα	1462	267	*S. cerevisiae* α factor signal sequence
Tags	1735	84	*c-myc*, FLAG and 6xHis tags
TMET15	1822	52	*S. cerevisiae MET15* terminator
PLEU2	2009	408	*S. cerevisiae LEU2* promoter
LEU2	2417	1095	*S. cerevisiae LEU2* CDS
2 µm	3956	1343	*S. cerevisiae* two micron plasmid replication origin
PTEF1	5321	387	*S. cerevisiae TEF1* promoter
PEM7	5715	48	*E. Coli* EM7 promoter
Sh ble	5781	375	*Streptoalloteichus hindustanus Sh ble* gene
CYC1	6221	248	*S. cerevisiae CYC1* transcription terminator
Ori	6543	589	*E. coli* pUC plasmid replication origin

(C)

Expected c-*myc* sequence:

End of signal sequence — *Pvu*II — c-*myc*

```
AAA AGA GAG GCT GAA GCT CAG CTG GAA CAA AAA TTG ATT TCT GAA GAA GAT TTG
 K   R   E   A   E   A   Q   L   E   Q   K   L   I   S   E   E   D   L
```

c-*myc* sequence in plasmid pYEDIS:

End of signal sequence — *Pvu*II — c-*myc*

```
AAA AGA GAG GCT GAA GCT CAG CTG GAA CAA AAT TGA TTTCTGAAGAAGATTTG
 K   R   E   A   E   A   Q   L   E   Q   N  Stop
```

Fig. S4. Map and features of the pYEDIS vector. (A) Vector map. The location of all primers used to generate the five PCR fragments that were assembled *in vitro* to generate the pYEDIS plasmid (see Material and Methods) are shown as red arrows. Note the location of the single *Pvu*II restriction site used to linearize the plasmid and introduce the GOI, as well as the two *Not*I restriction sites used to remove the *GAL1* promoter prior to transformation of *P. pastoris* YEDIS. Semi-circular light-blue arrows correspond to the regions of the plasmid that were sequenced, and displayed the expected sequence with the exception of the indicated new stop codon (STOP*) discussed in the text. (B) Location, length and description of all the elements in the pYEDIS vector. (C) Direct sequencing of plasmid pYEDIS revealed that the expected five consecutives adenines in codons 1 and 2 of c-*myc* (in red) were actually four adenines, changing the reading frame and causing the appearance of the new stop codon (STOP* in panel (A)).

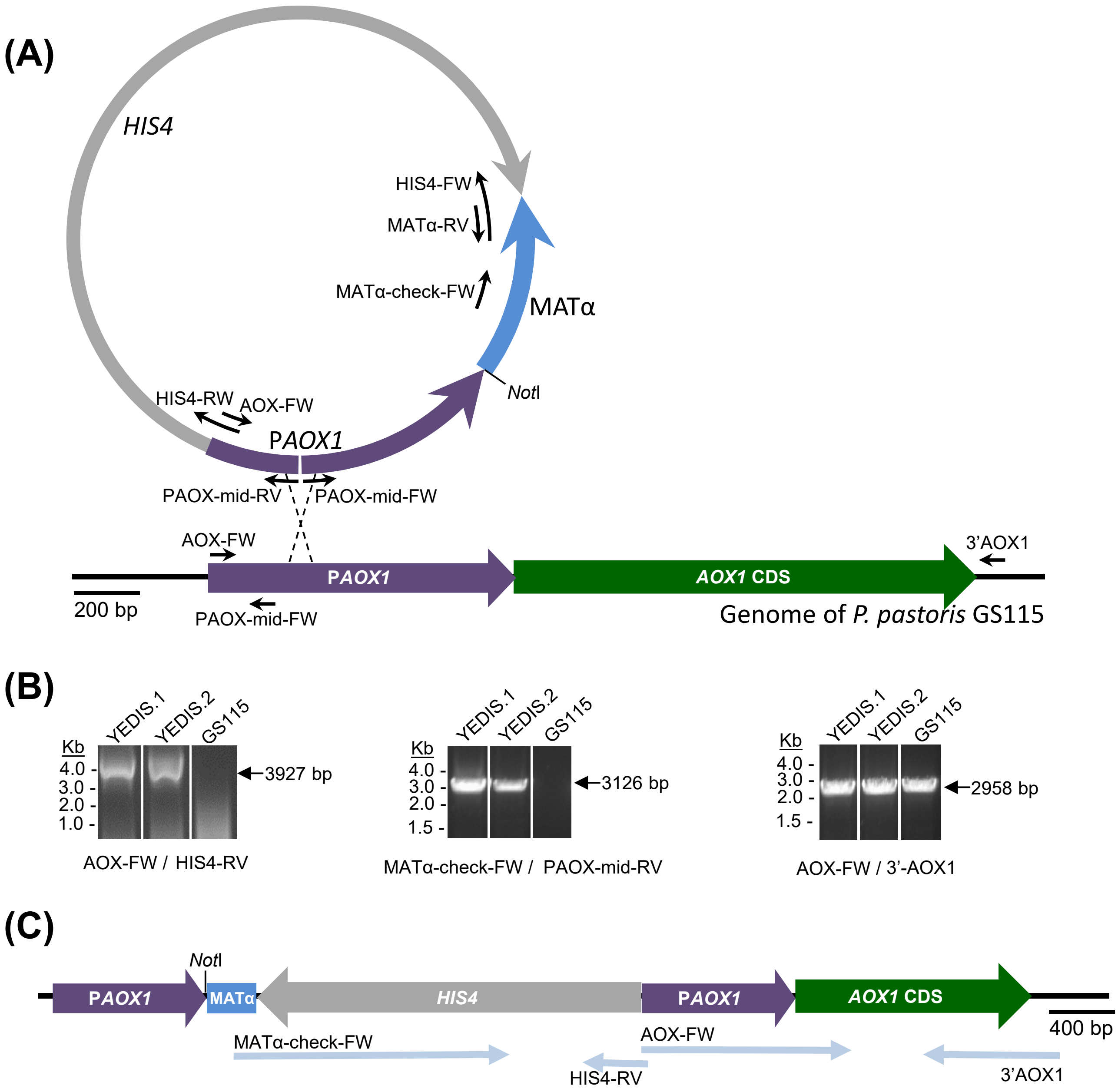

Fig. S5. Generation of *P. pastoris* YEDIS. (A) A DNA fragment containing the *P. pastoris AOX1* promoter (P*AOX1*) and the α factor signal sequence (MATα), amplified with primers AOX-FW and MATα-RV, was fused *in vitro* by its two ends with a second PCR product carrying the *P. pastoris HIS4* gene, amplified with primers HIS4-FW and HIS4-RV. Fusion was possible by the ends introduced by the primers HIS4-FW and HIS4-RV, homologous to the α factor signal sequence and the *HIS4* gene respectively. The resulting circular molecule was then amplified with primers PAOXmid-FW and PAOXmid-RV, resulting in the depicted linear DNA fragment used to transform *P. pastoris* GS115. Integration of the transforming DNA into P*AOX1* results in the *P. pastoris* YEDIS strain. (B) Three PCR reactions were carried out to analyse the correct genomic integration of the transforming DNA in the transformants. PCR products obtained for two positive transformants (YEDIS.1 and YEDIS.2) or the parental strain (GS115), using the indicated primers, are shown. (C) Scheme of the *P. pastoris* YEDIS genome, showing the *Not*I restriction site designed to be the site of integration for the pYEDIS plasmid (see text for details). The PCR products described in (B) were partially sequenced as indicated by light blue arrows marked with the primers used in the sequencing reactions.

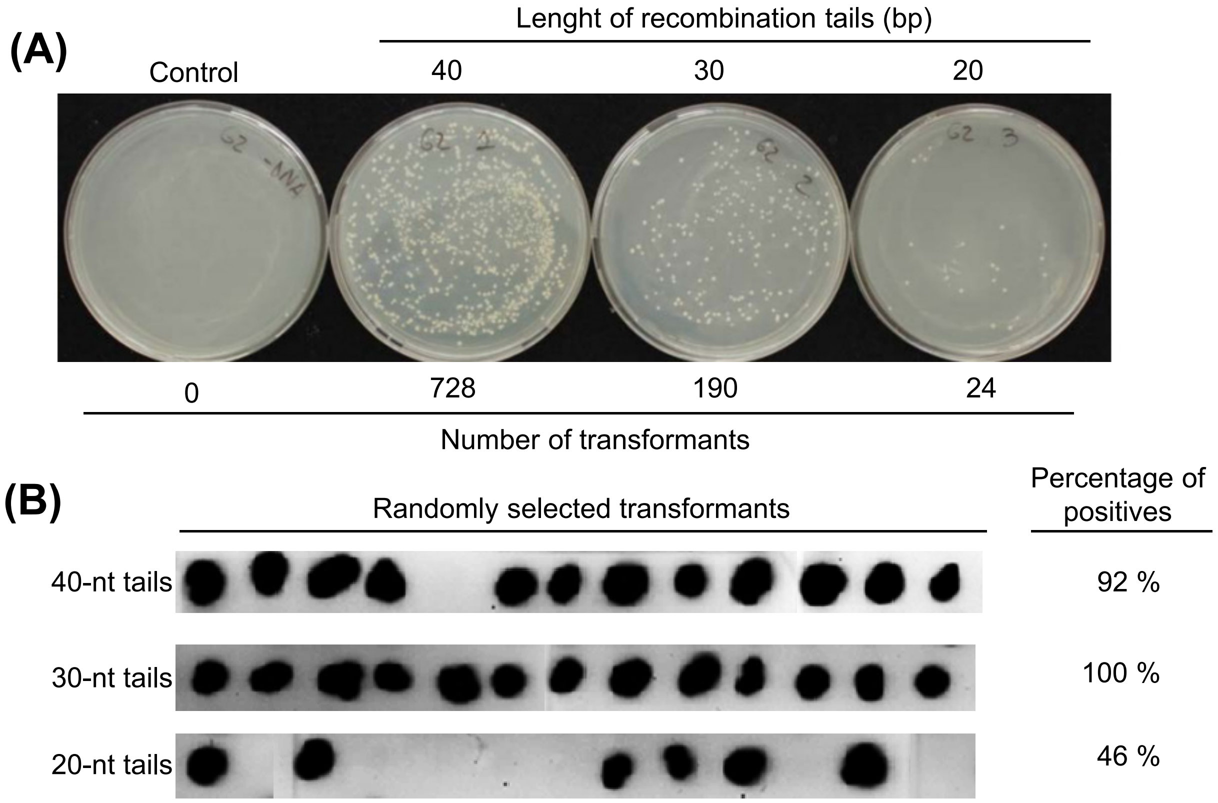

Fig. S6. Effect of the length of recombination tails on the frequency of transformants expressing the *B. cinerea* protein BcIEB1. (A) Effect of the length of the tails on the number of transformants obtained. *S. cerevisiae* YEDIS-G2 was co-transformed with the *Bcieb1* gene, flanked by recombination tails for the indicated length, and with the Fragment B. In the control, the *Bcieb1* gene was omitted from the transformation mix, but the Fragment B was maintained. (B) Colony blot screening of transformants for BcIEB1 expression. Thirteen randomly chosen colonies from each transformation were screened by colony blot with anti-c-*myc* antibodies as explained in the legend to Figure 2a.

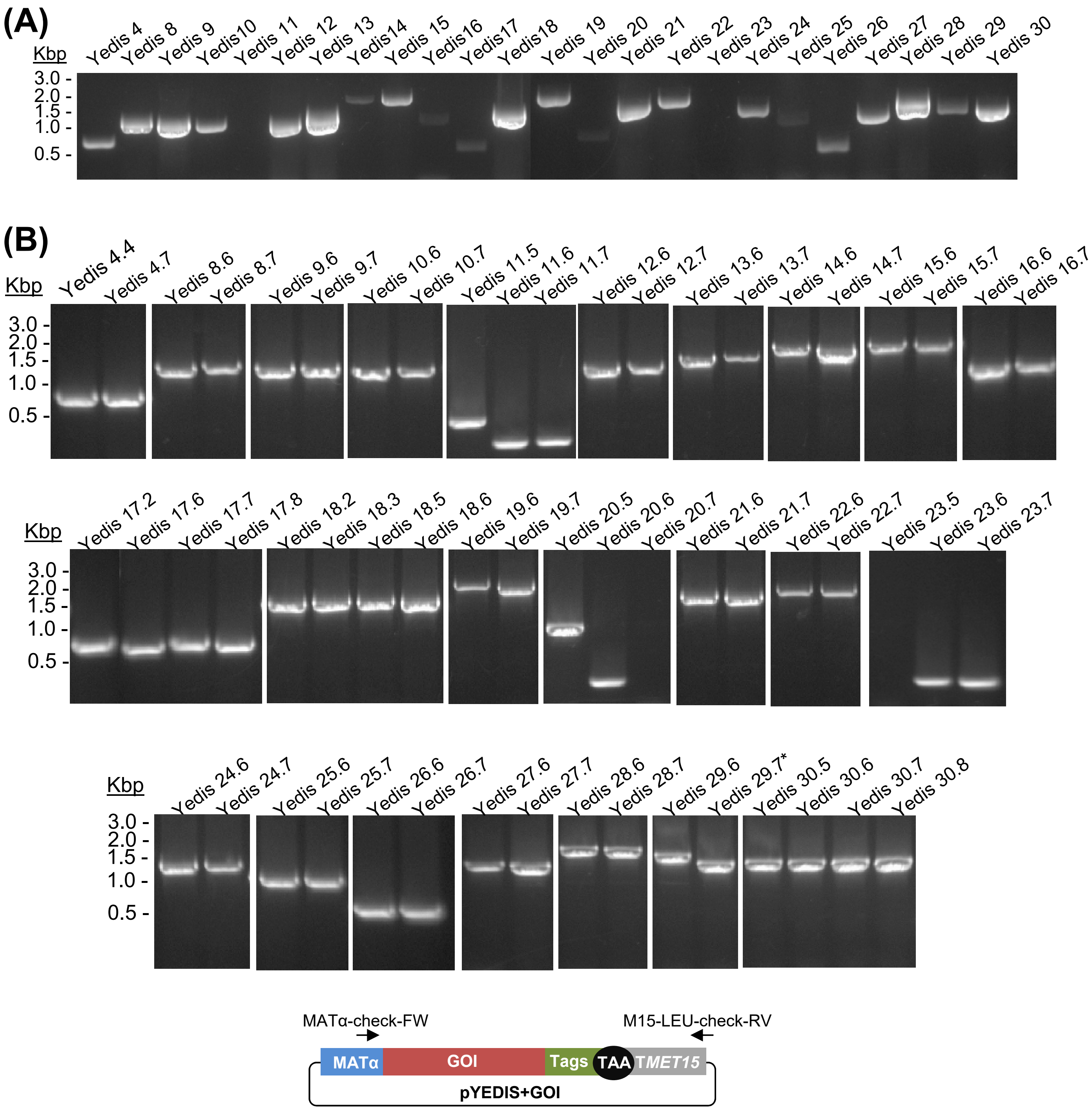

Fig. S7. High-throughput expression of *B. cinerea* proteins in *S. cerevisiae* using the pYEDIS vector. (A) PCR amplification of 23 *B. cinerea* genes (see details in table S3) with primers including 30-nt recombination tails (Table S2) and using 50 ng of *B. cinerea* cDNA as template. (B) Colony PCR with primers MATα-check-FW y M15-LEU-check-RV of 2-4 colonies obtained by co-transformation of *S. cerevisiae* BY4741 with *Pvu*II-digested pYEDIS vector and the amplicons shown in (A). The scheme indicates primer binding sites.
